# Global, regional, and national prevalence and disability-adjusted life-years for infertility in 195 countries and territories, 1990–2017: results from a global burden of disease study, 2017

**DOI:** 10.18632/aging.102497

**Published:** 2019-12-02

**Authors:** Hui Sun, Ting-Ting Gong, Yu-Ting Jiang, Shuang Zhang, Yu-Hong Zhao, Qi-Jun Wu

**Affiliations:** 1 Department of Clinical Epidemiology, Shengjing Hospital of China Medical University, Shenyang, China; 2 Clinical Research Center, Shengjing Hospital of China Medical University, Shenyang, China; 3 Department of Obstetrics and Gynecology, Shengjing Hospital of China Medical University, Shenyang, China

**Keywords:** female infertility, male infertility, prevalence, disability-adjusted life-years, global burden of disease study

## Abstract

To provide comprehensive estimates of the global, regional, and national burden of infertility from 1990 to 2017, using findings from a 2017 study on the global burden of disease (GBD), we assessed the burden of infertility in 195 countries and territories from 1990 to 2017. DisMod-MR 2.1 is a Bayesian meta-regression method that estimates non-fatal outcomes using sparse and heterogeneous epidemiological data. Globally, the age-standardized prevalence rate of infertility increased by 0.370% per year for females and 0.291% per year for males from 1990 to 2017. Additionally, age-standardized disability-adjusted life-years (DALYs) of infertility increased by 0.396% per year for females and 0.293% per year for males during the observational period. An increasing trend to these burden estimates was observed throughout the all socio-demographic index (SDI) countries. Interestingly, we found that high SDI countries had the lowest level of prevalence and DALYs in both genders. However, the largest increasing trend was observed in high-SDI countries for females. By contrast, low-SDI countries had the largest increasing trend in males. Negative associations were observed between these burden estimates and the SDI level. The global disease burden of infertility has been increasing throughout the period from 1990 to 2017.

## INTRODUCTION

Infertility is the inability to conceive within 1 year of unprotected intercourse, and it has been identified as a public health priority [1]. The Centers for Disease Control and Prevention of the United States emphasizes that infertility is more than a quality-of-life issue, with considerable public health consequences including psychological distress, social stigmatization, economic strain, and marital discord [2, 3]. Globally, infertility affects 15% of couples of reproductive age [4, 5]. A report from the 2006–2010 National Survey of Family Growth estimated that 6% of married females aged 15–44 years in the United States are infertile, and 12% have impaired fecundity, defined as the inability to conceive and carry a baby to term [6]. By contrast, among couples of reproductive age in China, the prevalence of infertility was 25% [7]. Furthermore, infertility is associated with increased risk of subsequent chronic health conditions such as cardiovascular disease [5].

A woman who is unable to bear a child is classified as having primary infertility. A woman who has previously conceived and successfully given birth yet is unable to do so subsequently is classified as having secondary infertility. Using survey data from 277 demographic and reproductive health surveys a study showed differences in the prevalence of primary and secondary infertility between 1990 and 2010 in 190 countries and territories [8]. Some regions have a high prevalence of primary infertility, but a low prevalence of secondary infertility, such as North Africa and the Middle East, notably Morocco and Yemen. However, some areas have a high prevalence of secondary infertility but a low prevalence of primary infertility, such as Central and Eastern Europe and Central Asia. Additionally, several previous studies provided information regarding the prevalence of infertility according to sex. For example, the reported prevalence of infertility in Britain was 12.5% among females but 10.1% among males [9]. Of note, among these published studies, some focused only on females [10–12]. Others exclusively examined males registered at infertility clinics [13, 14]. As such, these studies were based on relatively small groups, unrepresentative of the larger population of infertile people [15, 16].

Infertility affects both sexes across the globe. On a global scale, accurate information regarding the burden of infertility is sorely lacking. Without accurate national and regional data on infertility, it is impossible to identify and comprehensively treat infertile patients. Therefore, in this systematic analysis, we assessed the global burden of infertility from 1990 to 2017 based on prevalence and disability-adjusted life-years (DALYs), and we assessed its relationship to the level of development, using the socio-demographic index (SDI; a composite indicator of income per person, years of education, and fertility).

## RESULTS

### Infertility prevalence

**Figure 1:**
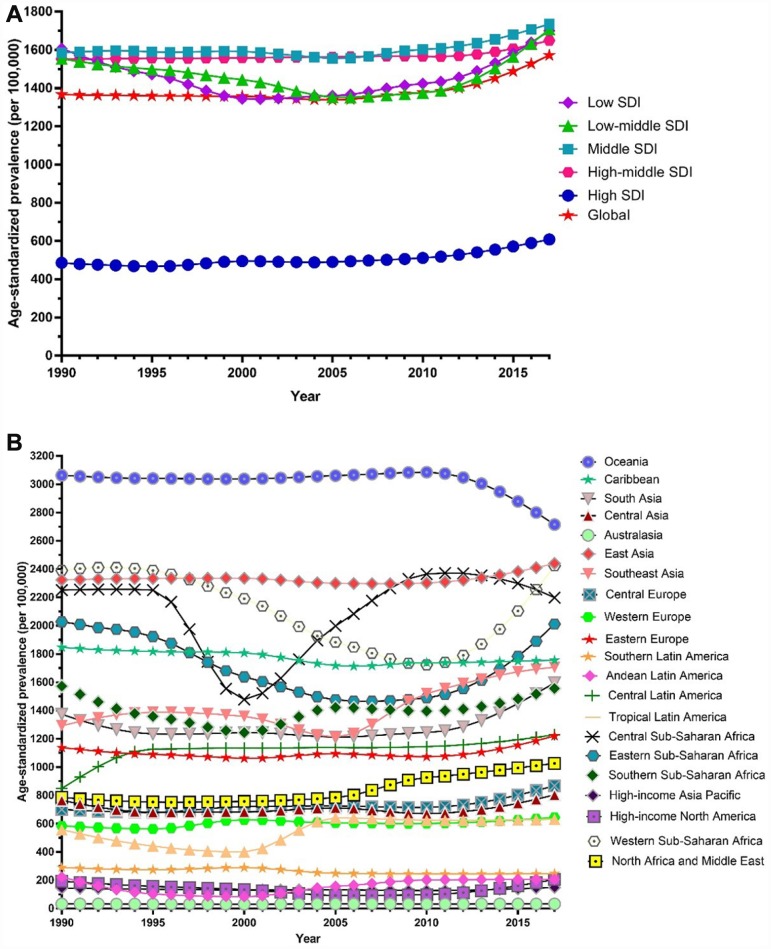
**Trends in global disease burden of female infertility prevalence from 1990–2017.** (**A**) Trends in global disease burden of female infertility prevalence by socio-demographic index from 1990–2017; (**B**) Trends in global disease burden of female infertility prevalence by region from 1990–2017).

**Figure 2:**
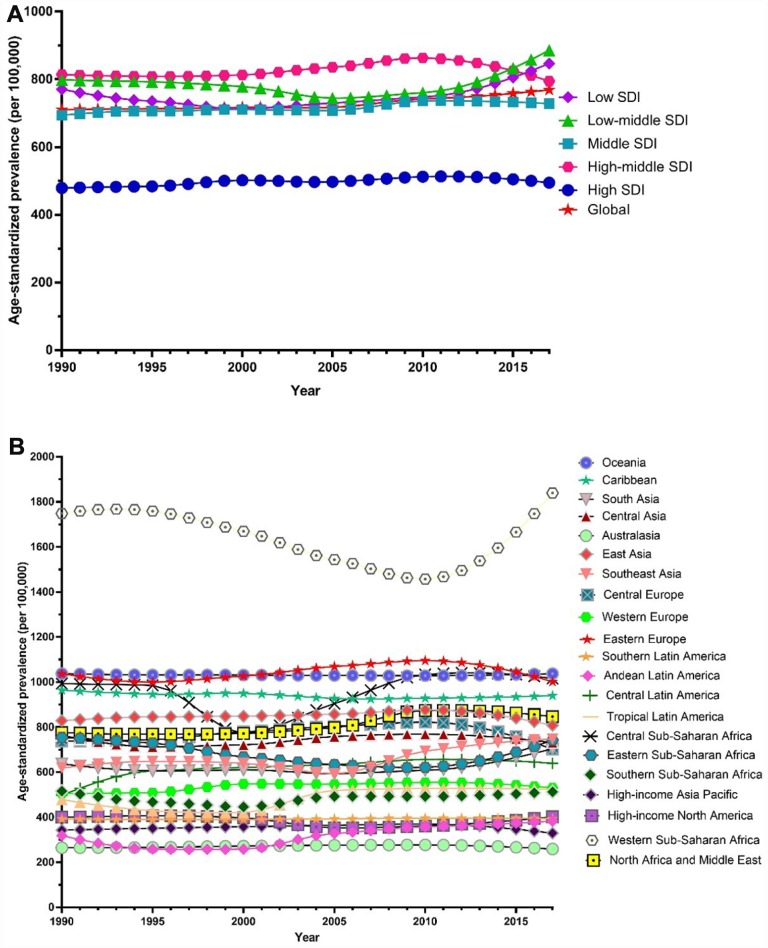
**Trends in global disease burden of male infertility prevalence from 1990–2017.** (**A**) Trends in global disease burden of male infertility prevalence by socio-demographic index from 1990–2017; (**B**) Trends in global disease burden of male infertility prevalence by region from 1990–2017).

**Figure 3:**
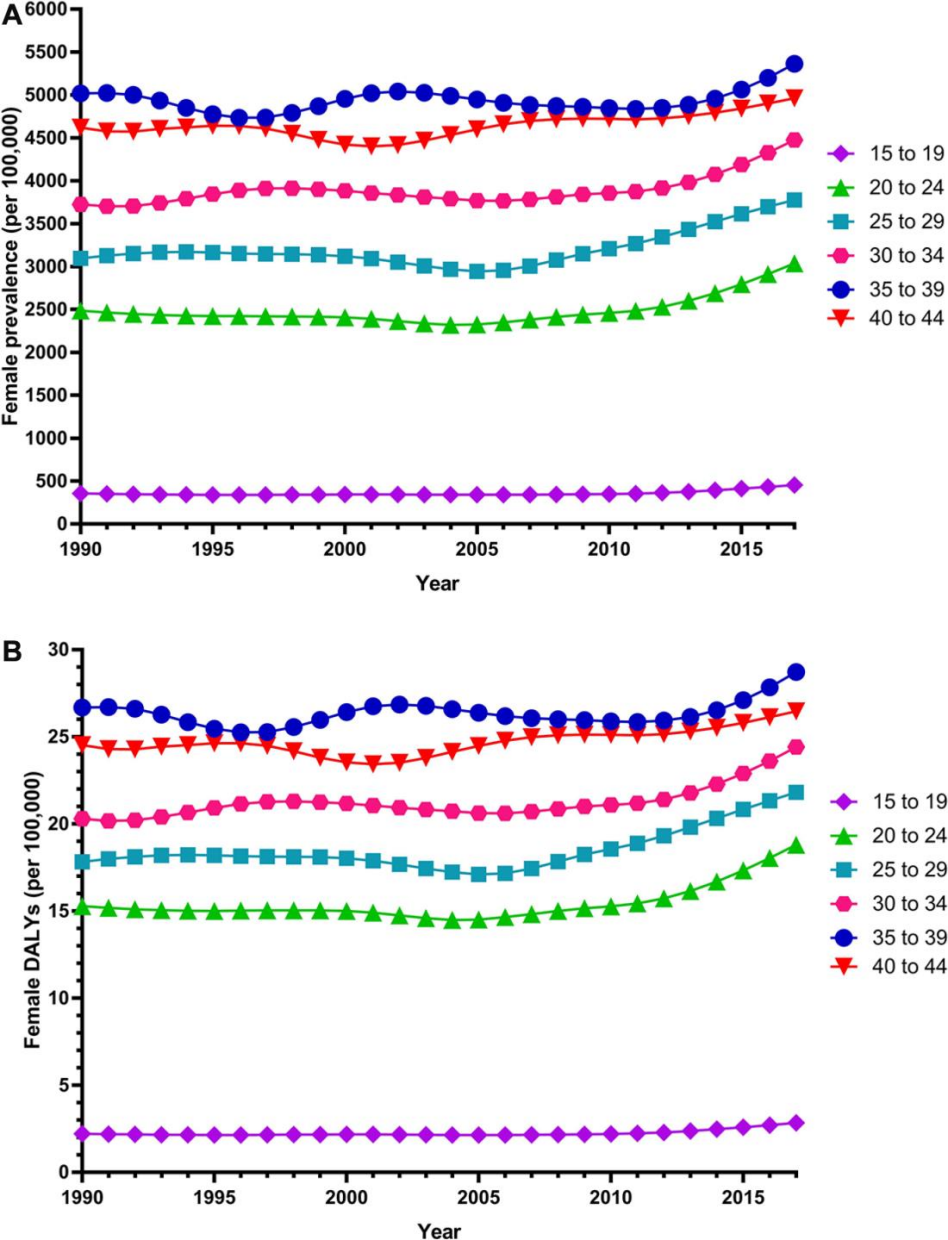
**Trends in global disease burden of 15–44 year-old female infertility prevalence and DALYs from 1990–2017.** (**A**) Prevalence; (**B**) DALYs).

**Figure 4:**
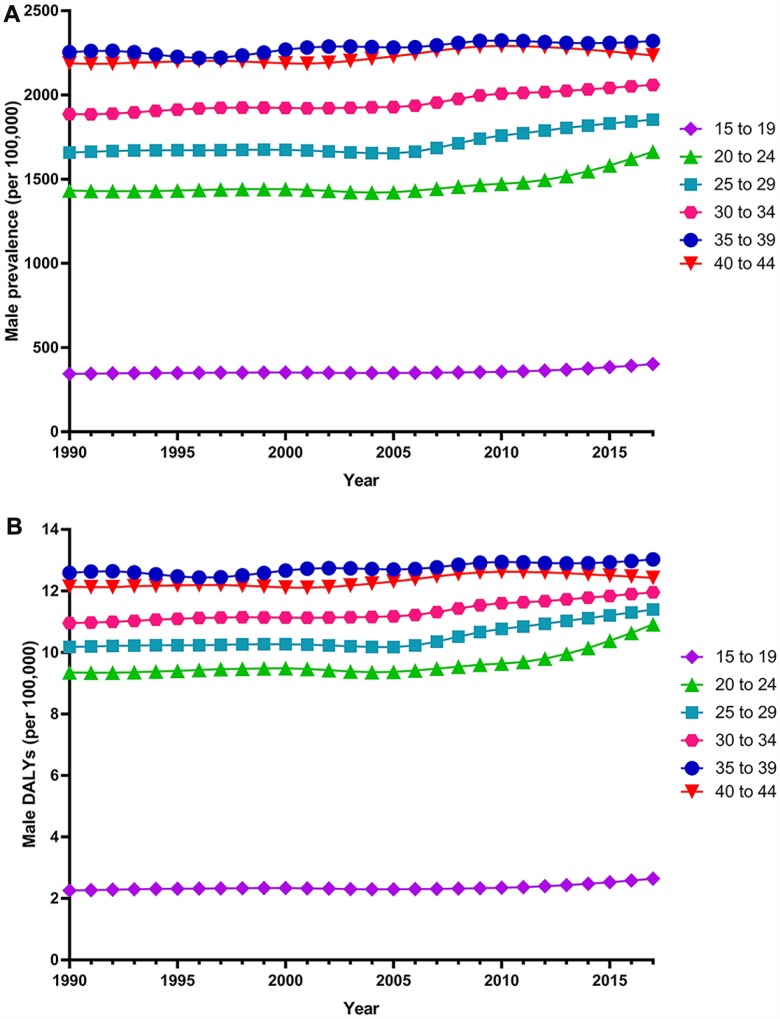
**Trends in global disease burden of 15–44 year-old male infertility prevalence and DALYs from 1990–2017.** (**A**) Prevalence; (**B**) DALYs).

Globally, the age-standardized prevalence rate of female infertility increased by 14.962% from 1366.85 per 100,000 (95% UI: 988.34, 1819.86) in 1990 to 1571.35 per 100,000 (95% UI: 1115.30, 2121.94) in 2017, representing a shift of 0.370% per year (95% CI: 0.213, 0.527) (Figure 1). The age­standardized prevalence rate of male infertility increased by 8.224% from 710.19 per 100,000 (95% UI: 586.08, 848.94) in 1990 to 768.59 per 100,000 (95% UI: 623.20, 929.91) in 2017, with an increasing rate of 0.291% per year (95% CI: 0.241, 0.341) (Figure 2). Among those aged 15–44 years in 2017, the 35–39 age group had the highest prevalence rate, and the 15–19 age group had the lowest (Figures 3 and 4). When stratified by SDI quintiles, we observed an increasing trend in all SDI countries. Of note, although high-SDI countries had the lowest prevalence rate throughout the observational period among both genders (Figures 1 and 2), the high-SDI quintile had the largest increasing trend (annual percentage change (APC) = 0.766%) in females, with a 51.41% contribution rate to the total increasing trend (Supplementary Tables 1 and 2). By contrast, low-SDI countries had the largest increasing trend (APC = 0.385%) in males, with a 33.75% contribution rate to the total increasing trend (Supplementary Tables 1 and 2).

Among females, 14 regions showed an increasing trend among the 21 regions (Figure 1). The largest APC was observed in Andean Latin America (2.129%), followed by Tropical Latin America (1.504%) and North Africa and the Middle East (1.352%), which contributed 53.78% to the overall increasing trend (Supplementary Tables 1 and 2). Among males, increasing trends were observed in 16 of the 21 regions (Figure 2). The largest APC was detected in Andean Latin America (1.558%), followed by Tropical Latin America (0.926%) and Southeast Asia (0.660 %), which contributed 47.39% to the overall increasing trend (Supplementary Tables 1 and 2).

**Figure 5:**
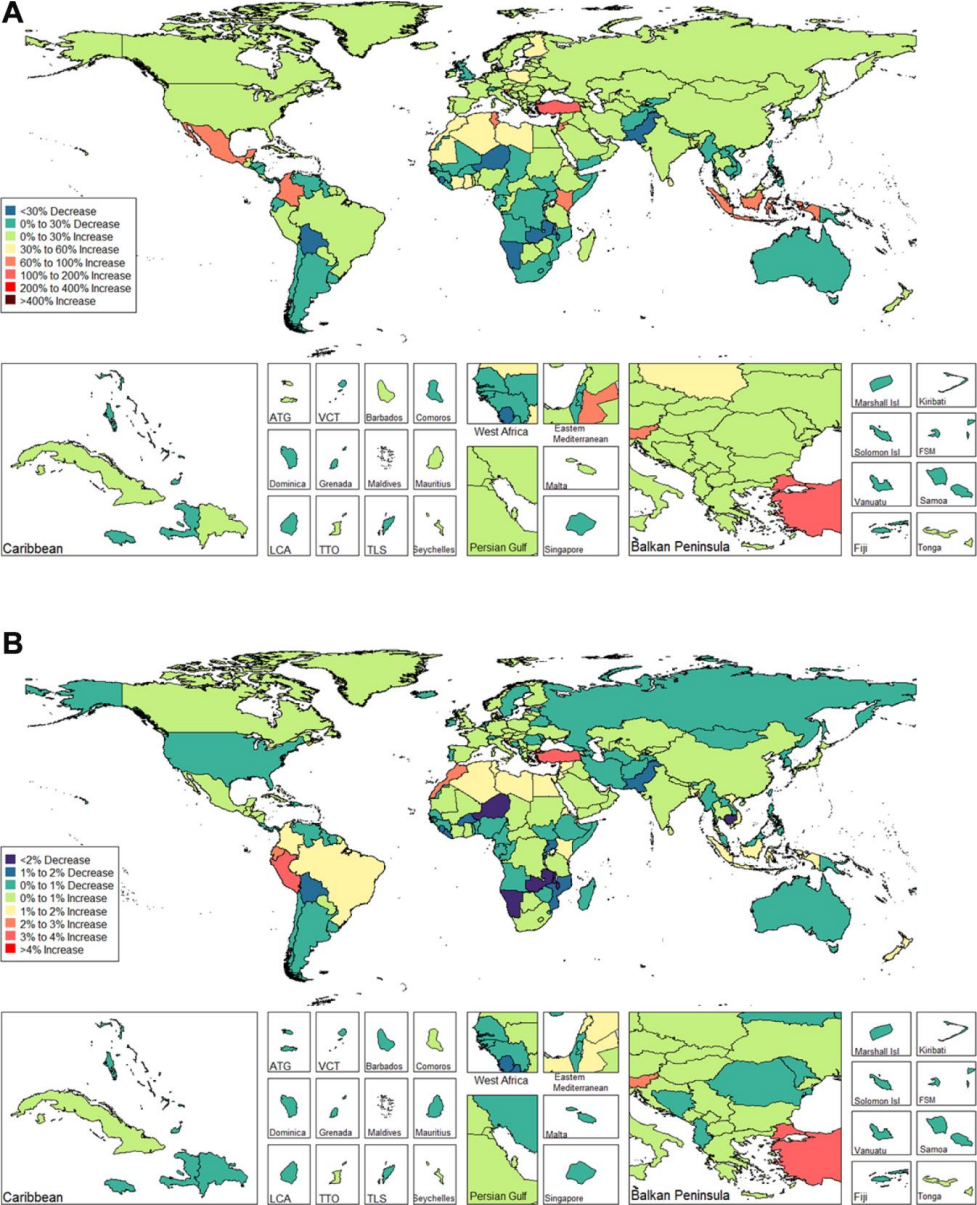
**Global disease burden of female infertility prevalence in 195 countries and territories.** (**A**) The percent change in age-standardized prevalence of female infertility between 1990 and 2017; (**B**) The estimated annual percentage change of female infertility age-standardized prevalence from 1990 to 2017).

**Figure 6:**
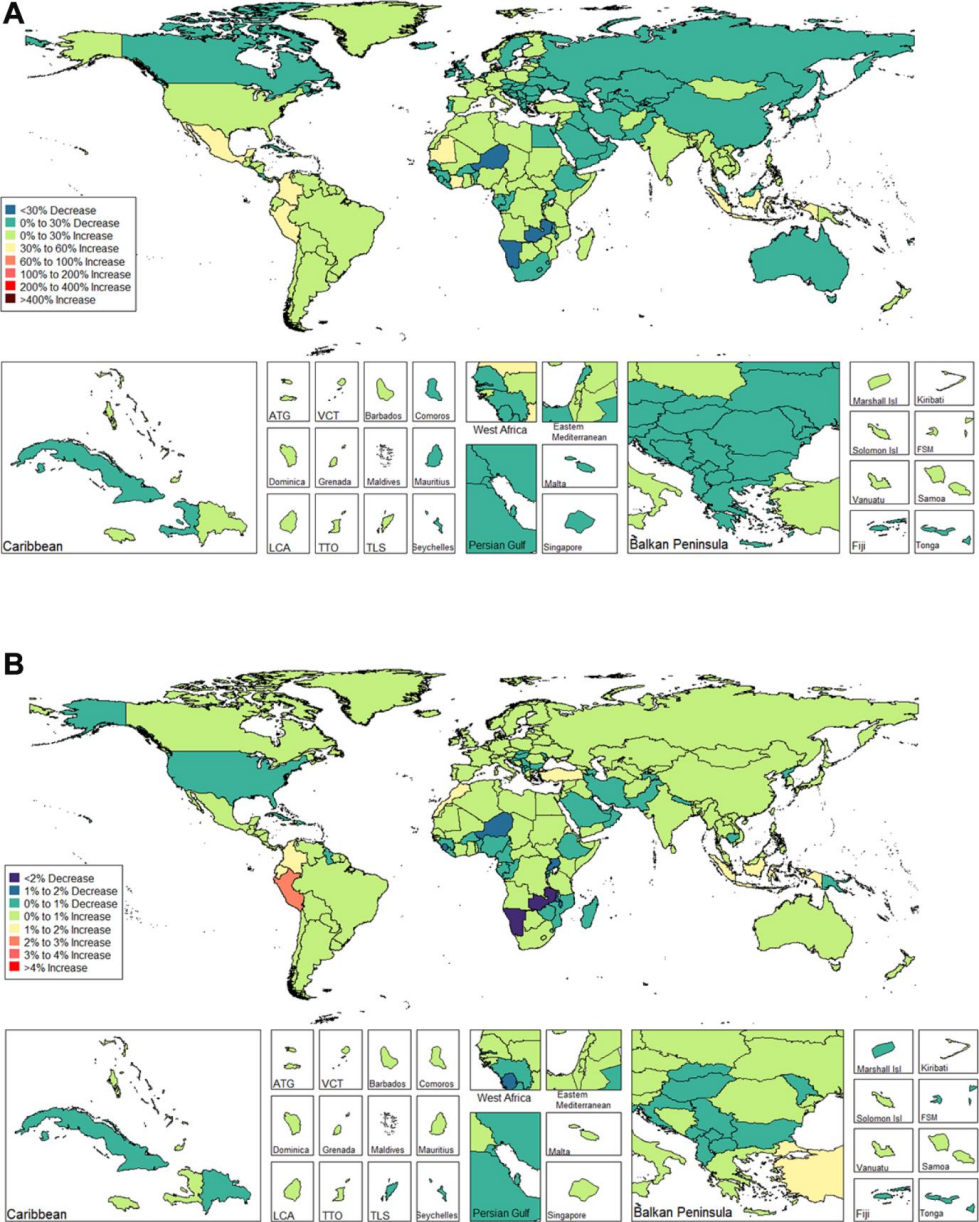
**Global disease burden of male infertility prevalence in 195 countries and territories.** (**A**) The percent change in age-standardized prevalence of male infertility between 1990 and 2017; (**B**) The estimated annual percentage change of male infertility age-standardized prevalence from 1990 to 2017).

We observed an increasing age-standardized prevalence of infertility among 89 and 136 countries and territories for females and males, respectively (Figures 5 and 6 and Supplementary Table 3). Among females, the top three countries and territories with increasing trends were Turkey (3.928%), Peru (3.597%), and Morocco (2.711%) (Figure 5 and Supplementary Table 3). By contrast, the top three countries and territories with decreasing trends were Zambia (-5.954%), Namibia (-5.943%), and Burundi (-3.112%) (Figure 5 and Supplementary Table 3). Among males, the top three countries and territories with increasing trends were Peru (2.265%), Morocco (1.676%), and Turkey (1.498%) (Figure 6 and Supplementary Table 3). By contrast, the top three countries and territories with decreasing trends were Zambia (-2.900%), Namibia (-2.181%), and Niger (-1.750%) (Figure 6 and Supplementary Table 3).

### Infertility DALYs

**Figure 7:**
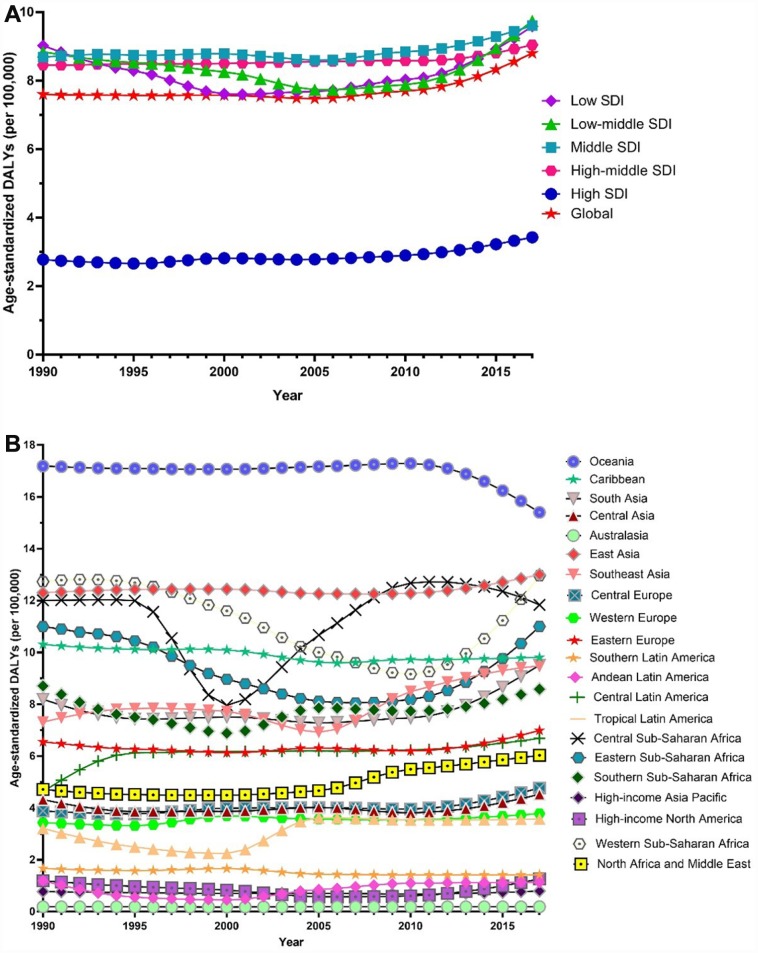
**Trends in global disease burden of female infertility disability-adjusted life-years from 1990–2017.** (**A**) Trends in global disease burden of female infertility disability-adjusted life-years by socio-demographic index from 1990–2017; (**B**) Trends in global disease burden of female infertility disability-adjusted life-years by region from 1990–2017).

**Figure 8:**
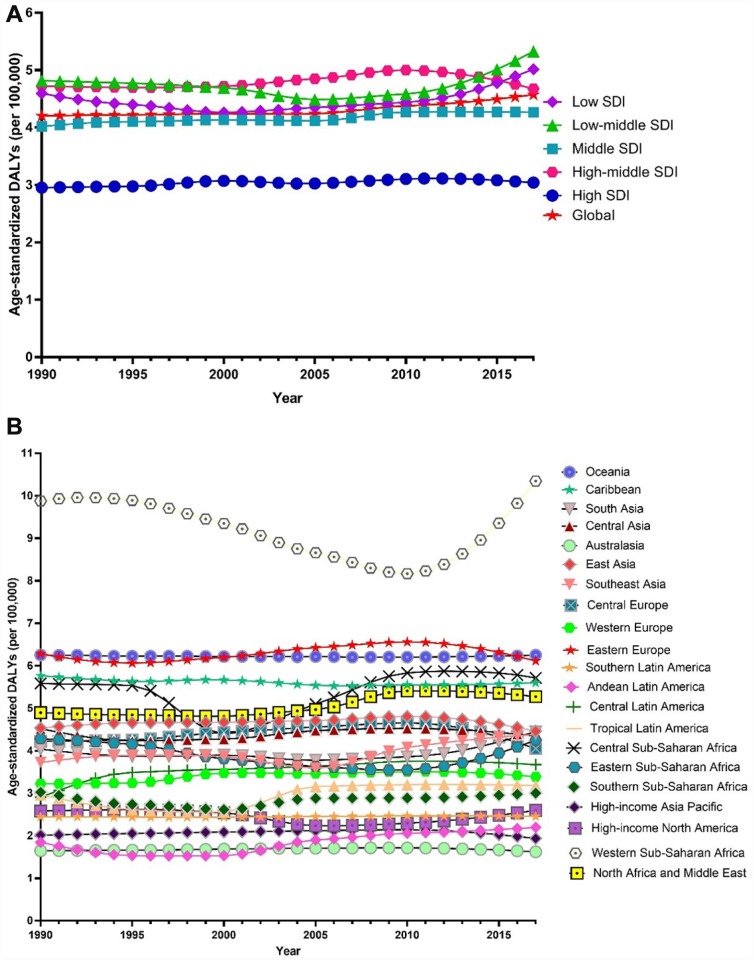
**Trends in global disease burden of male infertility disability-adjusted life-years from 1990–2017.** (**A**). Trends in global disease burden of male infertility disability-adjusted life-years by socio-demographic index from 1990–2017; (**B**). Trends in global disease burden of male infertility disability-adjusted life-years by region from 1990–2017).

Globally, age-standardized DALYs of female infertility increased by 15.834% from 7.599 per 100,000 (95% UI: 2.881, 15.974) in 1990 to 8.802 per 100,000 (95% UI: 3.328, 18.539) in 2017, at 0.396% per year (95% CI: 0.239, 0.552) (Figure 7). The age-standardized DALYs of male infertility increased by 8.843% from 4.20 per 100,000 (95% UI: 1.75, 8.75) in 1990 to 4.57 per 100,000 (95% UI: 1.89, 9.45) in 2017, at 0.293% per year (95% CI: 0.237, 0.349) (Figure 8). Among those aged 15–44 years in 2017, the 35–39 age group had the highest DALYs, and the 15–19 age group had the lowest (Figures 3 and 4). When stratified by SDI quintiles, we observed an increasing trend in all SDI countries (Figures 7 and 8). Of note, although high-SDI countries had the lowest prevalence rate throughout the observational period among both genders (Figures 1 and 2), the high-SDI quintile had the largest increasing trend (annual percentage change (APC) = 0.714%) in females, with a 46.95% contribution rate to the total increasing trend (Supplementary Tables 4 and 5).

Among females, an increasing trend was observed in 14 of the 21 regions (Figure 7). Similar to prevalence, Andean Latin America (2.200%), Tropical Latin America (1.487%) and North Africa and the Middle East (1.273%) were the top three regions, contributing 54.34% to the overall increasing trend (Supplementary Tables 4 and 5). Among males, we observed an increasing trend in 16 of the 21 regions (Figure 8). The top three regions were Andean Latin America (1.436%), Tropical Latin America (0.871%), and Central Latin America (0.543%), contributing 46.97% to the overall increasing trend (Supplementary Tables 4 and 5).

**Figure 9:**
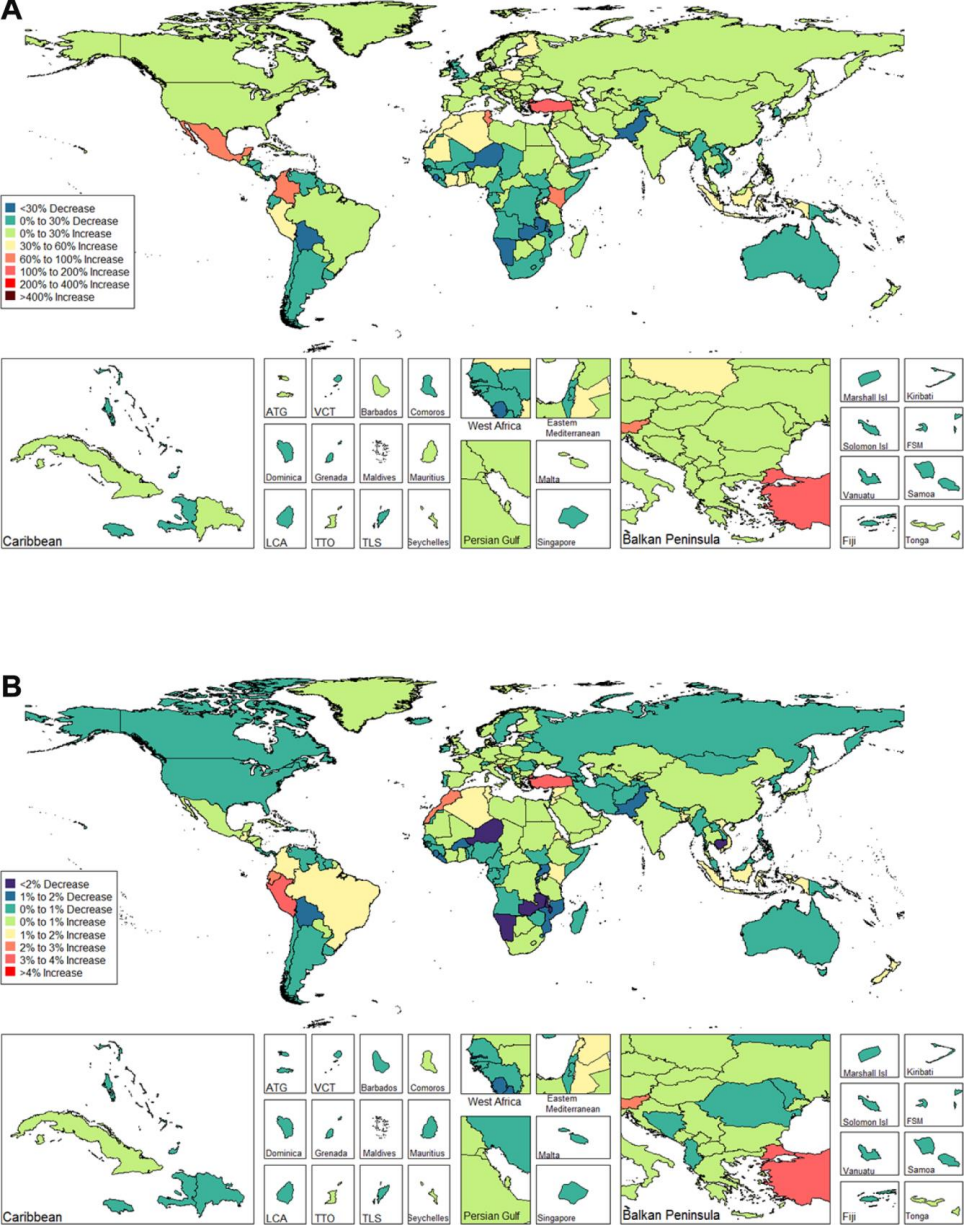
**Global disease burden of female infertility disability-adjusted life-years in 195 countries and territories.** (**A**). The percent change in age-standardized disability-adjusted life-years of female infertility between 1990 and 2017; (**B**) The estimated annual percentage change of female infertility age-standardized disability-adjusted life-years from 1990 to 2017).

**Figure 10:**
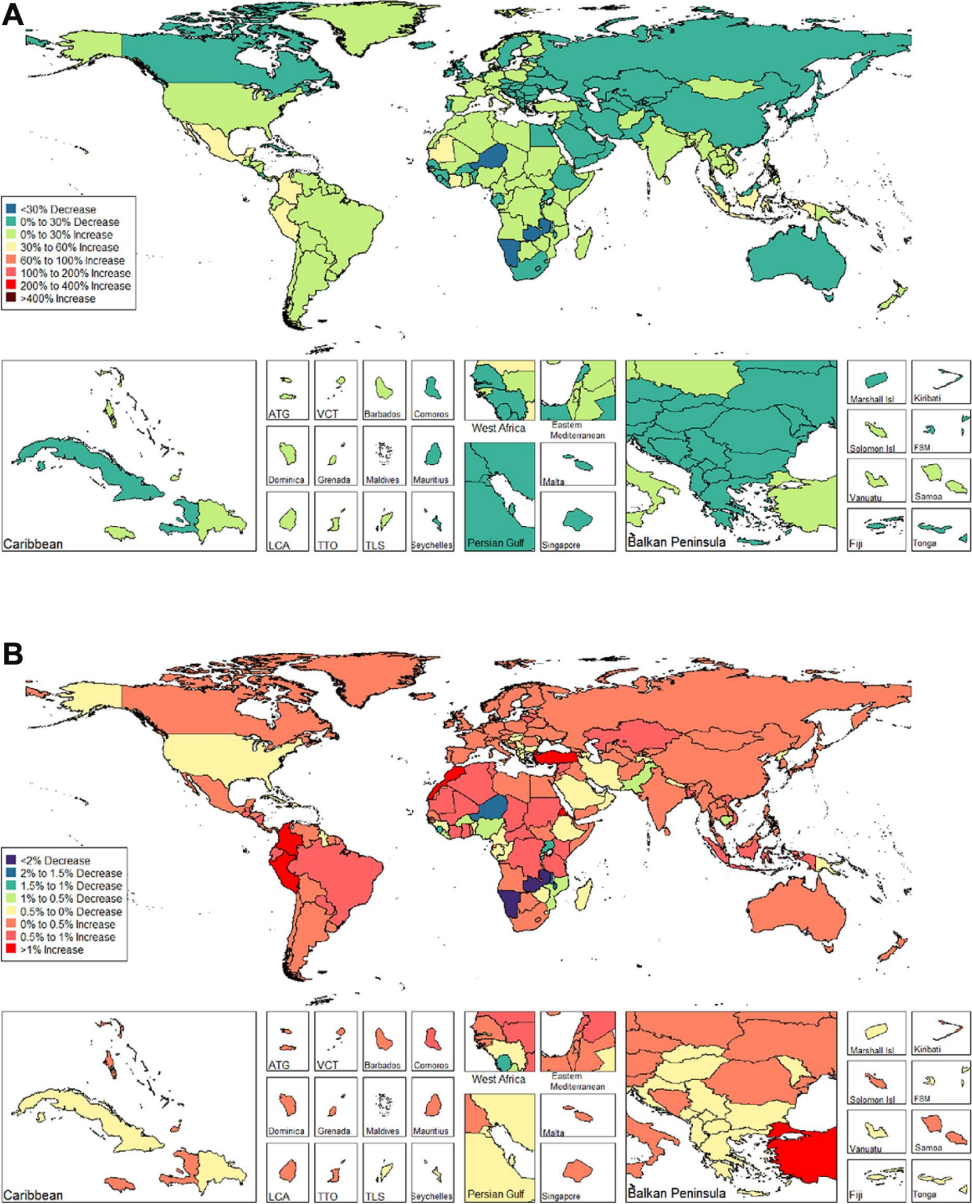
**Global disease burden of male infertility disability-adjusted life-years in 195 countries and territories.** (**A**). The percent change in age-standardized disability-adjusted life-years of male infertility between 1990 and 2017; (**B**). The estimated annual percentage change of male infertility age-standardized disability-adjusted life-years from 1990 to 2017).

We observed increasing age-standardized DALYs of infertility among 87 and 132 countries and territories for females and males, respectively (Figures 9 and 10, and Supplementary Table 6). Among females, the top three countries that increased were Turkey (3.667%), Peru (3.659%), and Morocco (2.772%) (Figure 9 and Supplementary Table 6). In contrast, the top three countries that decreased were Zambia (-5.842%), Namibia (-5.783%) and Burundi (-2.973%) (Figure 9 and Supplementary Table 6). Among males, the top three countries that increased were Peru (2.091%), Morocco (1.671%), and Turkey (1.326%) (Figure 10 and Supplementary Table 6). In contrast, the top three countries that decreased were Zambia (-2.863%), Namibia (-2.216 %), and Niger (-1.843 %) (Figure 10 and Supplementary Table 6).

### Global burden estimates of infertility in relation to SDI levels

**Figure 11:**
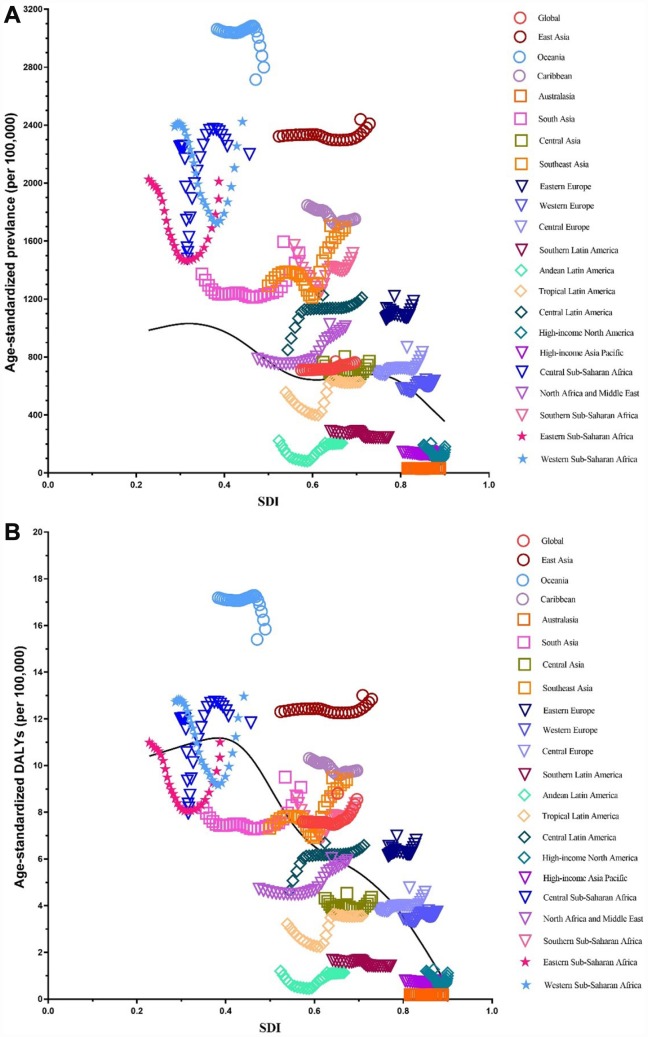
**Co-evolution of age-standardized burden estimates with SDI globally and for GBD regions for female infertility from 1990–2017.** (**A**). Prevalence (**B**) DALYs. Colored lines show global and region values for age-standardized burden estimates rates. Each point in a line represents 1 year starting at 1990 and ending at 2017. The black line represents the average expected relationship between SDI and burden estimates rates for female infertility based on values from each region in the 1990–2017 estimation period. DALYs = disability-adjusted life-years. SDI = Socio-demographic Index.

**Figure 12:**
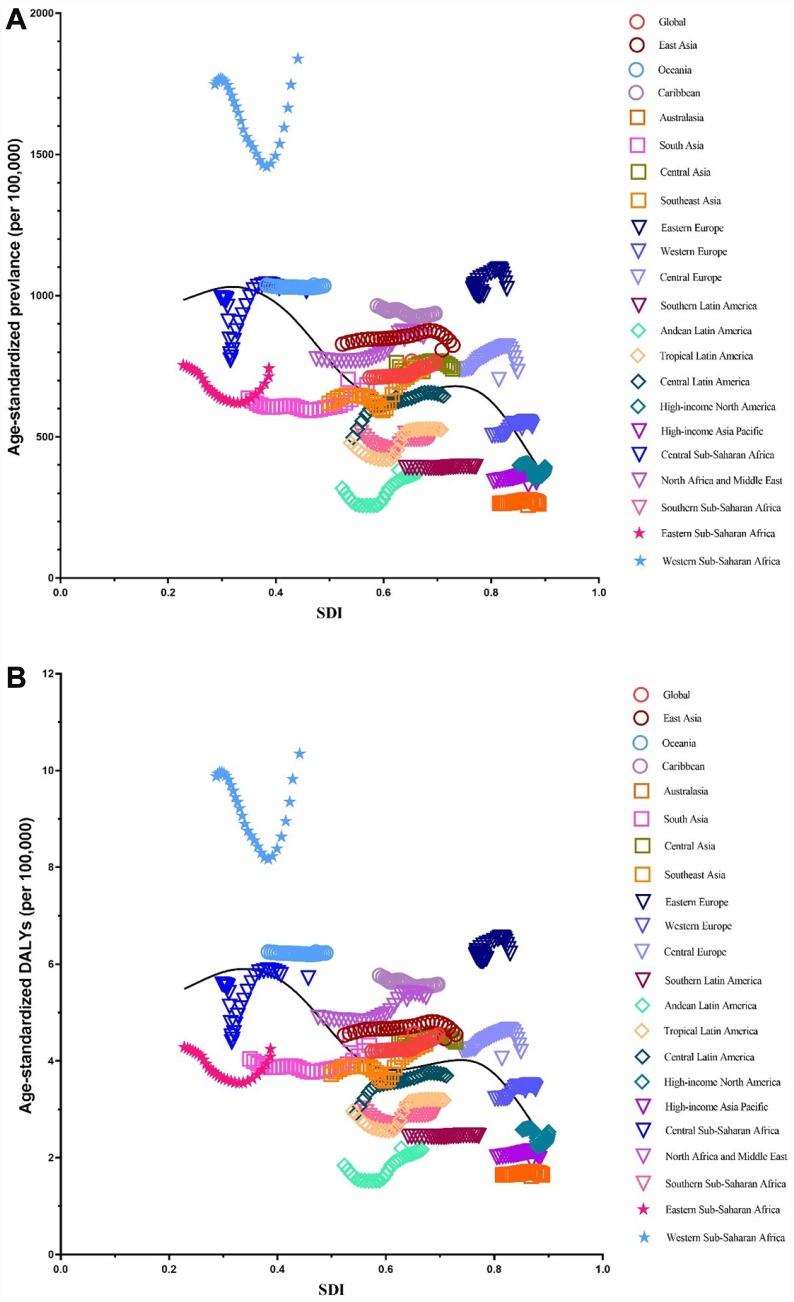
**Co-evolution of age-standardized burden estimates with SDI globally and for GBD regions for male infertility 1990–2017.** (**A**) Prevalence (**B**) DALYs. Colored lines show global and region values for age-standardized burden estimates rates. Each point in a line represents 1 year starting at 1990 and ending at 2017. The black line represents the average expected relationship between SDI and burden estimates rates for male infertility based on values from each region in the 1990–2017 estimation period. DALYs = disability-adjusted life-years. SDI = Socio-demographic Index.

We illustrated the associations between global burden estimates of infertility and the SDI levels for each of the 21 global burden of disease (GBD) regions for all individual years between 1990 and 2017 (Figures 11 and 12). General negative associations were observed between burden estimates and the SDI level. In brief, burden estimates tended to be stable when the SDI was limited to < 0.4. Subsequently, when the SDI was over 0.4, we observed negative associations between burden estimates and the SDI level. For Western Sub-Saharan Africa, we observed a U-shape association between prevalence and DALYs, and the SDI level. Similar patterns were observed in the Eastern and Central Sub-Saharan Africa.

## DISCUSSION

To the best of our knowledge, this is the first study to provide a comprehensive assessment of the values and trends of burden estimates of infertility by sex in 195 countries and territories from 1990 to 2017 on the basis of GBD 2017 [17, 18]. The burden estimates of male and female infertility, as measured by prevalence and DALYs, increased globally between the observational period, and it increased in all countries regardless of the SDI. Of note, we observed the largest increasing burden estimates in low-SDI countries for males but in high-SDI countries for females. We expect that our findings will be invaluable to health professionals toward their efforts to reduce the burden of infertility in their respective regions.

This study demonstrated that the prevalence of female infertility is relatively higher than that of male infertility. However, limited studies have focused on infertility by gender. Nevertheless, our findings are consistent with these studies [9, 19]. Meanwhile, an etiological study that included community-based females and their husbands or male partners and clinically-based patients showed that risk factors accounted for 65.9% of female infertility etiology, whereas this number was a mere 6.8% for male infertility [19]. It can be seen that the potential for infertility in females is greater than it is in males. The reason why the prevalence of female infertility is higher than male infertility might be attributed to two reasons. First, unlike female infertility, male infertility is not well reported in general, especially in countries where cultural differences and patriarchy prevent accurate statistics from being collected and compiled. Second, a study has shown that tubal factor infertility was the most common cause [19]. Reproductive health is of special importance to females, particularly during their reproductive years. Males also have reproductive health concerns and needs, but their general health is affected by their reproductive health to a lesser extent than in females [20]. Infertility caused by female reproductive health problems is more common. This helps to explain why the prevalence of infertility in females is higher than in males.

Among global infertile females and males aged 15–44 years from 1990 to 2017, the 35–39 age group had the highest prevalence and the 15–19 age group had the lowest. Researchers estimated the cumulative incidence of infertility for 1,037 males and females using a longitudinal birth cohort study in Dunedin, New Zealand. The results showed that the most pronounced incidence of infertility occurred during the mid- to late-30s [21]. In another study, researchers analyzed data from the infertility component of the 2009–2010 Canadian Community Health Survey for married and common-law couples with a female partner aged 18–44. Couples with lower parity (0 or 1 child) had significantly higher odds of being infertile when female partners were aged 35–44 years, compared to those 18–34 years old [22]. Another cross-sectional population survey showed that the age-adjusted odds of experiencing infertility were significantly higher among females who first gave birth at age 35 or older compared with those who did so before the age of 25 [9]. A similar, though slightly weaker, association was observed among males. These studies are very similar to our results. As far as we know, age at marriage can play an important role in causing infertility [23]. Over the past decades, conjugal unions have been delayed, resulting in couples starting to live together or getting married at an older age. This has led to a delay in childbearing, with females being older when first attempting pregnancy. A quantitative cross-sectional survey showed that a longer duration of infertility is associated with a significant decrease in the live-birth rate [24]. Meanwhile, females in their mid- to late-30s are nearing the end of their reproductive spans, when males may be experiencing an age-related decline in fertility. Because patients are older, the disease is more serious and the success rate of treatment is lower. Moreover, younger patients are prioritized for publicly funded infertility treatment in countries such as New Zealand [23, 24]. As such, older patients have less access to treatment.

We found that the largest increasing burden estimates were in low-SDI countries for males and in high-SDI countries for females. This may be attributed to the increasing rate of infertility detection, especially in males with low SDI levels, due to the gradual development of national economies. Of note, high-SDI countries had the lowest prevalence rate for both sex. To the best of our knowledge, disparities in infertility are likely due to differential distributions of factors such as education, socioeconomic status, health behavior, access to quality infertility services, and service-seeking behavior. Studies in Europe, North America, and Australia show that the large majority of research participants who experienced infertility but did not seek medical help. This is of concern, as are the marked inequalities in seeking help among those who are well qualified and employed in high-status jobs compared to those who are not [25–27]. A study has shown that the proportion of couples seeking medical care was 56% in developed countries and 51% in developing countries [28]. Although it is not possible to treat all these couples successfully, treatment will lead to a decline in infertility rates in economically developed regions. Thus, we found the lowest prevalence in areas with high-SDI countries. It is quite surprising that Datta et al. found that infertility was most common among females with a post-secondary degree and lowest among those with no academic qualifications, whereas no statistically significant association was observed among males in this regard. A large body of literature describes a trend among females in developed countries of delaying procreation, and it is expected that this changing tempo to fertility is becoming a global phenomenon [29]. Meanwhile, with overall improvements to the economy and changes to lifestyle, the number of overweight (and underweight) individuals is increasing, where obesity is an important factor leading to infertility [30]. Esmaeilzadeh et al. found in their study that infertile females had a 4.8-fold increased risk of obesity and an almost 3.8-fold increased risk of being overweight compared to fertile females [31].

Our investigation has several strengths. First, to the best of our knowledge, this is the first comprehensive overview of the epidemiological situation and trends regarding the female and male infertility burden around the world. Second, the GBD 2017 [17, 18] approach to estimating the prevalence of infertility is novel and can be repeated with relative efficiency. Our findings will be useful to resource allocation and health services planning for the growing number of patients with infertility. However, GBD 2017 [17, 18] methods have several limitations. First, data are absent or extremely sparse for some regions of the world. As such, the models we used to predict prevalence and DALYs might lead to unusual changes in segments of the data. We cannot ignore that the relatively low burden of infertility in developing countries is related to the under-diagnosis of the condition due to limited access to specialized medical care, imaging resources, and laboratory investigations. Until such information becomes available, however, we maintain that the results from our model are valid. Second, the data lacks robust predictive covariates for infertility to aid in population-based risk assessments. GBD is actively seeking access to medical claims data in other countries to improve the accuracy of estimates for diseases such as infertility, for which every patient can be expected to be in contact with the health­care system if there are no major barriers to accessing care. Through our network of collaborators, we expect that future iterations of GBD will be able to add such sources from other countries. Third, there is no relevant data on risk factors of infertility in the GBD database. As such, we cannot compare the magnitude of the risk factors for infertility. Finally, reports on intentional injuries (especially self-harm and legal intervention) are subject to underreporting or even being covered up in many countries. Many of the countries involved in conflicts do not have a reliable health information system even in their preconflict states. We did not evaluate the indirect effects of collective violence (war) on total population. For example, Africa is affected by war, political and economic instability, resulting in population decrease [32, 33].

In summary, the burden estimates of infertility increased globally for both genders between 1990 and 2017. This report provides an integrated, contemporary understanding of the global infertility disease burden. Our findings can inform policymakers regarding the health care priority of infertility, and preventive and managerial interventions must be implemented to address the growing burden of infertility in these regions. More studies are needed to investigate the risk factors of infertility in order to carry out efficient preventive and managerial strategies to reduce the burden of this disease.

## METHODS

### Data sources

The Global Burden of Diseases, Injuries, and Risk Factors Study, 2017 (GBD 2017) employed a standardized analytical method that used all eligible sources to estimate epidemiological data, including prevalence and DALYs, for 354 causes by sex, age, and location from 1990 to 2017 (17). It estimated all parameters for 195 countries and territories, nested in 21 regions. Details of the methodology of GBD studies and the main changes applied in GBD 2017 are provided in other articles (see supplementary file 1) [17, 18].

### Modeling

For GBD 2017, the following case definitions were used for infertility: primary infertility was defined as existing in a couple who have not had a live birth, who wanted a child, and had been in a relationship for more than 5 years without using contraceptives. Secondary infertility was defined as existing in a couple who wanted a child and have been in a relationship for more than 5 years without using contraceptives since a previous live birth. Estimation was completed in three steps [17]. First, we estimated the total primary and secondary infertility in couples. This was accomplished by first quantifying the rate of infertility among married survey respondents and then quantifying how this married population related to the overall population. Second, we modeled the proportion of primary and secondary infertility due to female and male factors, respectively, to estimate four “envelopes” of infertility: male primary infertility, male secondary infertility, female primary infertility, and female secondary infertility. Third, we executed a “causal attribution” process to assign cases of each envelope to likely underlying causes and assigned the remainder to idiopathic infertility. Non-fatal modeling, using DisMod-MR 2.1, was performed to estimate the prevalence of infertility [34]. DisMod-MR 2.1 is a Bayesian meta-regression method that estimates non-fatal outcomes using sparse and heterogeneous epidemiological data. It also pools data from different sources, adjusts them for variations in study methods across sources, and enforces consistency between different epidemiological parameters. Binary study-level covariates were used to minimize the residual errors of the estimated prevalence and years lived with disability (YLD). Using mixed-effects nonlinear regression on all the available data at the global level, super-region Bayesian priors were generated; likewise, the super-region regression model was then used to generate regional Bayesian priors, and so on down the cascade [34, 35]. YLD were calculated by multiplying the prevalence of each sequela by its disability weight and adding the procedure-related morbidity associated with infertility treatment [34]. Years of life lost (YLL) due to infertility were calculated using normative global life expectancy. DALYs were calculated by summing the YLD and YLL [36].

### Socio-demographic Index

The SDI is a summary measure that estimates a location’s position on a spectrum of development. The SDI and epidemiological transition SDI is a summary measure that places all GBD locations on a spectrum of socioeconomic development [37]. SDI, expressed on a scale of 0 to 1, is a summary measure that identifies where GBD locations sit on the spectrum of socioeconomic development [37]. The SDI is calculated based on the geometric mean of lag-distributed income, average years of schooling among populations aged 15 years or older, and total fertility rate. More details regarding the calculation of the SDI are provided in previous GBD publications [17, 18, 38]. All 195 countries and territories were then categorized into five regions in terms of the SDI; low, low-middle, middle, high-middle, and high. The cutoff values used to determine quintiles for analysis were then computed using country-level estimates of SDI for 2017, excluding countries with populations of less than 1 million. These quintiles are used to categorise and present GBD 2017 results on the basis of sociodemographic status. Additional details on and results from the SDI calculation are available in the supplementary file (Supplementary Table 1)

### Statistical analysis

We ran DisMod-MR 2.1 models to estimate the proportion of primary and secondary infertility by sex, proportion of primary female infertility, proportion of secondary female infertility, proportion of primary male infertility, and proportion of secondary male infertility. We model sex-specific infertility as a proportion [17]. Prevalence was estimated for nine impairments, defined as sequelae of multiple causes for which better data were available to estimate the overall occurrence than for each underlying cause: Infertility and eight other diseases [17]. We assumed that infertility does not lead to mortality and, therefore, DALYs of infertility are equal to their YLD [34]. So we used the age-standardized prevalence rate and DALYs as well as the annual percentage change (APC) to quantify female and male infertility burden estimated trends [39]. Restricting the age range to 15 to 44 years and divided six 5-year age groups. All measures were age-standardized using the GBD standard population. The age-standardized rates (per 100,000 people) in accordance with a direct method were calculated by summing the products of age-specific rates and the number of individuals in the same age subgroup of the selected reference standard population and subsequently dividing the sum of standard population weights. The APC is a widely used measure of trends in an age-standardized rate over a specific time interval. A regression line was fitted to the natural logarithm of the rates. The APC and 95% confidential interval (CI) values can also be obtained from a linear regression model [40, 41]. We employed a generalized additive model with locally estimated scatterplot smoothing to the SDI to estimate the associations between SDI and the age-standardized prevalence rate and DALYs using GBD estimates from all national locations from 1990 to 2017 [42]. All statistical analyses were performed using SPSS (Version 23, SPSS Inc.) and the R program, Version 3.4.4 (ggplot2, readxl, dplyr), with P values <.001 considered significant. R program Version 3.4.4 was used to generate figures of the final estimates of prevalence and DALYs from data available from ghdx. healthdata. org/ gbd- results- tool.

## Supplementary Material

Supplementary Materials

Supplementary Tables

Supplementary Table 3

Supplementary Table 6
