# Supplementary material for: Global, regional, and national prevalence and disability-adjusted life-years for infertility in 195 countries and territories, 1990–2017: results from a global burden of disease study, 2017
Source: Aging (Albany NY). 2019 Dec 2;11(23):10952–91. doi: 10.18632/aging.102497 (PMC6932903; doi:10.18632/aging.102497)
Supplement: Supplementary Materials [file aging-11-102497-s004..pdf]

SUPPLEMENTARY MATERIALS

GBD OVERVIEW

Geographic units of the analysis

The locations included in GBD 2017 have been arranged into a set of hierarchical categories composed of seven super-regions and a further nested set of 21 regions containing 195 countries and territories (Appendix Table 1). Subnational estimation in GBD 2017 includes Brazil, China, India, Indonesia, Japan, Kenya, Mexico, South Africa, Sweden, the United Kingdom, and the United States, and new subnational assessments at the administrative one level for Ethiopia, Iran, Norway, and Russia and by Maori ethnicity for New Zealand. For this publication, we present subnational estimates in figures only for all subnational countries with the exception of the new assessments which will be reported in separate publications. Combined, there are a total of 390 locations at the first subnational unit level. Included in subnational Level 1 locations are countries that have been subdivided into the first subnational level, such as states or provinces, for the GBD analysis; subnational Level 2 only applies to India, England, and Russia. For this paper we present data at the national and territory level.

Time period of the analysis

A complete set of cause-specific prevalence, and YLD numbers and rates were computed for the years 1990, 1995, 2000, 2005, 2010, and 2017. All GBD 2017 results and online data visualisations are available at http://vizhub.healthdata.org/gbd-compare1 with access to results for all GBD metrics.

Statement of GATHER compliance

This study complies with the Guidelines for Accurate and Transparent Health Estimates Reporting (GATHER) recommendations. We have documented the steps involved in our analytical procedures and detailed the data sources used in compliance with the GATHER.

The GATHER recommendations may be found here: http://gather-statement.org/

GBD results overview

Results from the Global Burden of Disease Study (GBD) are now measured in terabytes. Results are available in an interactive data downloading tool on the Global Health Data exchange (GHDx). Data and underlying code used for this analysis will be made publicly available pending manuscript acceptance.

The core summary results include years of life lost (YLLs), years lived with disability (YLDs), and disability-adjusted life-years (DALYs). The GHDx includes data for causes, risks, cause-risk attribution, aetiologies, and impairments.

Data input sources overview

GBD 2017 incorporated a large number and wide variety of input sources to estimate mortality, population, fertility, causes of death and illness, and risk factors for 195 countries and territories from 1990-2017. These input sources are accessible through an interactive citation tool available in the GHDx.

Users can retrieve citations for a specific GBD component, cause or risk, and geography by choosing from the available selection boxes. They can then view and access GHDx records for input sources and export a CSV file that includes the GHDx metadata, citations, and information about where the data were used in GBD. Additional metadata for each input source are available through the citation tool, as required by the GATHER statement.

Infertility Outcome estimation

Conceptually, the estimation effort is divided into eight major components: (1) compiling data sources through data identification and extraction; (2) data adjustment; (3) estimation of prevalence by cause and sequelae using DisMod-MR 2.1 or alternative modelling strategies for selected cause groups; (4) estimation by impairment; (5) severity distributions; (6) incorporation of disability weights; (7) comorbidity adjustment; and (8) the estimation of YLDs by sequelae and causes.

DATA SOURCES, IDENTIFICATION, AND EXTRACTION

Systematic reviews

For GBD 2017, we conducted literature reviews for 82 non-fatal causes and one impairment through February 2018. For other disease sequelae, only a small fraction of the existing data appears in the published literature and other sources predominate such as survey data, disease registers, notification data or hospital inpatient data. As was done in GBD 2016, data were systematically screened from household surveys archived in the Global Health Data Exchange (http://ghdx.healthdata.org/), including Demographic and Health Surveys, Multiple Indicator Cluster Surveys, Living Standards Measurement Surveys, and Reproductive Health Surveys.

Other national health surveys were identified based on survey series that had yielded usable data for past rounds of GBD, sources suggested to us by in-country collaborators, and surveys identified in major multinational survey data catalogs, such as the International Household Survey Network and the World Health Organization (WHO) Central Data Catalog, as well as through country Ministry of Health and Central Statistical Office websites. Case notifications reported to the WHO were updated through 2017. Citations for all data sources used for nonfatal estimation in GBD 2017 are provided in searchable form through a web tool (http://ghdx.healthdata.org/).

Survey data preparation

For GBD 2017, survey data for which we have access to the unit record data constitute a substantial part of the underlying data used in the estimation process. During extraction, we concentrate on demographic variables (such as location, sex, age), survey design variables (such as sampling strategy and sampling weights), and the variables used to define the population estimate (such as prevalence or a proportion) and a measure of uncertainty (standard error, confidence interval or sample size and number of cases).

Nonfatal disease registries

For GBD 2017 nonfatal estimation, disease registries were an important source for a select number of conditions such as cancers, end-stage renal disease, and congenital disorders.

Registry data is particularly key in the estimation of neoplasms given the increasing attention to noncommunicable diseases, particularly cancers, in low and middle-income areas of the world. The GHDx source tool (http://ghdx.healthdata.org/data-type/disease-registry) provides a comprehensive list of registry data used in GBD estimation processes.

Data adjustment

In addition to the corrections applied to claims and hospital data, a number of other adjustments were applied to extracted nonfatal sources in order to make the data more consistent and suitable for modelling. In this second step of nonfatal estimation, commonly applied adjustments included age-sex splitting, bias correction, adjustments for underreporting of notification data, and computing expected values of excess mortality. Age-sex splitting was commonly applied to literature data reported by age or sex but not by age and sex. For GBD 2017, we split all data reported in age groups with a width greater than 20 years, using age patterns from available survey microdata or regional patterns derived from an initial run of main modelling tool, DisMod-MR 2.1. We relied on the meta-regression component of DisMod-MR 2.1 for most of the bias correction of data for variations in study attributes such as case definitions and measurement method. DisMod-MR 2.1 calculates a single adjustment that is applied regardless of age, sex, or location. If enough data were available to differentiate these adjustments by age, sex, or location, or if detailed survey data were available to make more precise adjustments between different thresholds on a biochemical measure, we applied bias corrections to the data before entry into DisMod-MR 2.1. For instance, we crosswalked between 12 different case definitions with different thresholds of fasting plasma glucose or glycated hemoglobin levels for diabetes mellitus based on available survey data with individual records of the actual measurements. In another example, we corrected data on COPD from surveys applying different thresholds on spirometry measurements using studies that had reported on prevalence of COPD for the reference and alternative thresholds. As this relationship varied with age, age-specific correction factors were derived. The correction of notification data for underreporting relied on studies that had examined the gap between true incidence and notified cases.

IMPAIRMENT AND UNDERLYING CAUSE ESTIMATION

Impairments in GBD are conditions or specific domains of functional health loss which are spread across many GBD causes as sequelae and for which there are better data to estimate the occurrence of the overall impairment than for each sequela based on the underlying cause. Overall impairment prevalence was estimated using DisMod-MR 2.1. We constrained cause-specific estimates of impairments, as in the 19 causes of blindness, to sum to the total prevalence estimated for that impairment. Estimates were made separately for primary infertility (those unable to conceive), secondary infertility (those having trouble conceiving again), and whether the impairment affected men and/or women.

Disability weights

To compute YLDs for a particular health outcome in a given population, the number of people living with that outcome is multiplied by a disability weight that represents the magnitude of health loss associated with the outcome. Disability weights are measured on a scale from 0 to 1, with 0 implying a state that is equivalent to full health and 1 a state equivalent to death.

Disability weights used in GBD studies prior to GBD 2010 have been criticized for the method used (person trade-off), the small elite panel of international public health experts who determined the weights and the lack of consistency over time as the GBD cause list expanded and additional disability weights from a study in the Netherlands24 were added or others derived by ad-hoc methods.

YLD computation, uncertainty, and residual YLDs

For GBD 2017, we computed YLDs by sequela as prevalence multiplied by the disability weight for the health state associated with that sequela. The uncertainty ranges reported around YLDs incorporates uncertainty in prevalence and uncertainty in the disability weight. To do this, we take the 1,000 samples of comorbidity-corrected YLDs and 1,000 samples of the disability weight to generate 1,000 samples of the YLD distribution. We assume no correlation in the uncertainty in prevalence and disability weights. The 95% uncertainty interval is reported as the 25th and 975th values of the distribution. Uncertainty intervals for YLDs at different points in time (1990, 1995, 2000, 2005, 2010, and 2016) for a given disease or sequela are correlated because of the shared uncertainty in the disability weight. For this reason, changes in YLDs over time can be significant even if the uncertainty intervals of the two estimates of YLDs largely overlap as significance is determined by the uncertainty around the prevalence estimates.

Socio-demographic Index (SDI) analysis and epidemiological transition

The Socio-demographic Index (SDI) is a composite indicator of development status strongly correlated with health outcomes. In short, it is the geometric mean of 0 to 1 indices of total fertility rate under the age of 25 (TFU25), mean education for those aged 15 and older (EDU15+), and lag distributed income (LDI) per capita.

Development of revised SDI indicator

SDI was originally constructed for GBD 2015 using the Human Development Index (HDI) methodology, wherein a 0 to 1 index value was determined for each of the original three covariate inputs (total fertility rate in ages 15 to 49, EDU15+, and LDI per capita) using the observed minima and maxima over the estimation period to set the scales.

In response to feedback from collaborators and the evolution of the GBD, we have refined the indicator with each GBD cycle. For GBD 2017, in conjunction with our expanded estimation of age-specific fertility, we chose to replace the total fertility rate as one of the three component indices with the total fertility rate under 25 (TFU25). The TFU25 provides a better measure of womens status in society, as it focuses on ages where childbearing disrupts the pursuit of education and entrance into the workforce.

During GBD 2016 we moved from using relative index scales to absolute scales to enhance the stability of SDIs interpretation over time, as we noticed that the measure was highly sensitive to the addition of subnational units that tended to stretch the empirical minima and maxima. We selected the minima and maxima of the scales by examining the relationships each of the inputs had with life expectancy at birth and under-5 mortality and identifying points of limiting returns at both high and low values, if they occurred prior to theoretical limits (e.g., a TFU25 of 0).

Thus, an index score of 0 represents the minimum level of each covariate input past which selected health outcomes can get no worse, while an index score of 1 represents the maximum level of each covariate input past which selected health outcomes cease to improve. As a composite, a location with an SDI of 0 would have a theoretical minimum level of development relevant to these health outcomes, while a location with an SDI of 1 would have a theoretical maximum level of development relevant to these health outcomes.

The composite Socio-Demographic Index is the geometric mean of these three indices for a given location year. The cutoff values used to determine quintiles for analysis were then computed using country-level estimates of SDI for the year 2017.

The table below illustrates Socio-Demographic Index groupings by location, based on 2017 values

Location Name	2017 SDI Index Value	SDI Quintile
Global	0.652205351	
Central Europe, Eastern Europe, and Central Asia	0.765735064	
Central Asia	0.672778523	
Armenia	0.702021479	High-middle SDI

Azerbaijan	0.701169598	High-middle SDI
Georgia	0.699719344	High-middle SDI
Kazakhstan	0.735474229	High-middle SDI
Kyrgyzstan	0.606646902	Low-middle SDI
Mongolia	0.661854015	Middle SDI
Tajikistan	0.522612209	Low-middle SDI
Turkmenistan	0.696418617	Middle SDI
Uzbekistan	0.629546531	Middle SDI
Central Europe	0.813976167	
Albania	0.684614242	Middle SDI
Bosnia and Herzegovina	0.712609905	High-middle SDI
Bulgaria	0.79173721	High-middle SDI
Croatia	0.824844721	High SDI
Czech Republic	0.850980459	High SDI
Hungary	0.816804322	High-middle SDI
Macedonia	0.75436361	High-middle SDI
Montenegro	0.788188778	High-middle SDI
Poland	0.84377326	High SDI
Romania	0.784193905	High-middle SDI
Serbia	0.75179332	High-middle SDI
Slovakia	0.841690487	High SDI
Slovenia	0.860279598	High SDI
Eastern Europe	0.785420363	
Belarus	0.772665439	High-middle SDI
Estonia	0.857709406	High SDI
Latvia	0.825131484	High SDI
Lithuania	0.840877452	High SDI
Moldova	0.675572758	Middle SDI
Russian Federation	0.791738063	High-middle SDI
Ukraine	0.740061596	High-middle SDI
High-income	0.854428248	
Australasia	0.868509969	
Australia	0.873188291	High SDI
New Zealand	0.842273544	High SDI
High-income Asia Pacific	0.86894981	
Brunei	0.856240565	High SDI
Japan	0.865093512	High SDI
Aichi	0.874998978	High SDI
Akita	0.829009097	High SDI
Aomori	0.825175188	High SDI
Chiba	0.859238574	High SDI
Ehime	0.838399264	High SDI
Fukui	0.852281964	High SDI
Fukuoka	0.855307883	High SDI
Fukushima	0.830930555	High SDI
Gifu	0.84923591	High SDI

Gunma	0.850963336	High SDI
Hiroshima	0.862595627	High SDI
Hokkaido	0.841522308	High SDI
Hyogo	0.859765235	High SDI
Ibaraki	0.850665189	High SDI
Ishikawa	0.856039392	High SDI
Iwate	0.825241842	High SDI
Kagawa	0.849935485	High SDI
Kagoshima	0.829680279	High SDI
Kanagawa	0.874939342	High SDI
Kochi	0.825446834	High SDI
Kumamoto	0.831536501	High SDI
Kyoto	0.87256007	High SDI
Mie	0.853567757	High SDI
Miyagi	0.850313137	High SDI
Miyazaki	0.823112655	High SDI
Nagano	0.851209245	High SDI
Nagasaki	0.826141869	High SDI
Nara	0.847998888	High SDI
Niigata	0.843300137	High SDI
Oita	0.845989117	High SDI
Okayama	0.855866898	High SDI
Okinawa	0.817915416	High SDI
Osaka	0.872366437	High SDI
Saga	0.833665065	High SDI
Saitama	0.8520121	High SDI
Shiga	0.870844353	High SDI
Shimane	0.831040466	High SDI
Shizuoka	0.858790953	High SDI
Tochigi	0.853264467	High SDI
Tokushima	0.845285	High SDI
Tokyo	0.924328028	High SDI
Tottori	0.83436659	High SDI
Toyama	0.859824207	High SDI
Wakayama	0.839775092	High SDI
Yamagata	0.831923683	High SDI
Yamaguchi	0.849441807	High SDI
Yamanashi	0.854296098	High SDI
South Korea	0.871955704	High SDI
Singapore	0.872215248	High SDI
High-income North America	0.868169406	
Canada	0.882086227	High SDI
Greenland	0.760075292	High-middle SDI
United States	0.86662166	High SDI
Alabama	0.837233514	High SDI
Alaska	0.86060992	High SDI

Arizona	0.845107314	High SDI
Arkansas	0.826148933	High SDI
California	0.872398094	High SDI
Colorado	0.882128544	High SDI
Connecticut	0.906486727	High SDI
Delaware	0.873744053	High SDI
District of Columbia	0.890203139	High SDI
Florida	0.863631092	High SDI
Georgia	0.848426298	High SDI
Hawaii	0.872290363	High SDI
Idaho	0.840713155	High SDI
Illinois	0.879386003	High SDI
Indiana	0.84792909	High SDI
Iowa	0.8704793	High SDI
Kansas	0.864464964	High SDI
Kentucky	0.83130395	High SDI
Louisiana	0.834894869	High SDI
Maine	0.872309993	High SDI
Maryland	0.895667105	High SDI
Massachusetts	0.913307727	High SDI
Michigan	0.867717003	High SDI
Minnesota	0.892987345	High SDI
Mississippi	0.818942009	High SDI
Missouri	0.85325798	High SDI
Montana	0.863383139	High SDI
Nebraska	0.87308561	High SDI
Nevada	0.847315003	High SDI
New Hampshire	0.904304115	High SDI
New Jersey	0.899124902	High SDI
New Mexico	0.835274776	High SDI
New York	0.893442339	High SDI
North Carolina	0.84978326	High SDI
North Dakota	0.879820384	High SDI
Ohio	0.858271211	High SDI
Oklahoma	0.838181089	High SDI
Oregon	0.870700326	High SDI
Pennsylvania	0.878553277	High SDI
Rhode Island	0.890036984	High SDI
South Carolina	0.846024965	High SDI
South Dakota	0.860188872	High SDI
Tennessee	0.836985155	High SDI
Texas	0.837777472	High SDI
Utah	0.855766922	High SDI
Vermont	0.89559193	High SDI
Virginia	0.885122306	High SDI
Washington	0.88440099	High SDI

West Virginia	0.824706332	High SDI
Wisconsin	0.87773172	High SDI
Wyoming	0.869345173	High SDI
Southern Latin America	0.720171023	
Argentina	0.710150584	High-middle SDI
Chile	0.748081344	High-middle SDI
Uruguay	0.706753401	High-middle SDI
Western Europe	0.856820142	
Andorra	0.901838419	High SDI
Austria	0.866029424	High SDI
Belgium	0.886479194	High SDI
Cyprus	0.86457342	High SDI
Denmark	0.917864091	High SDI
Finland	0.892872363	High SDI
France	0.864667258	High SDI
Germany	0.869902009	High SDI
Greece	0.816993531	High SDI
Iceland	0.907023083	High SDI
Ireland	0.882181159	High SDI
Israel	0.81594436	High-middle SDI
Italy	0.843401161	High SDI
Luxembourg	0.915748227	High SDI
Malta	0.835898842	High SDI
Netherlands	0.911855053	High SDI
Norway	0.910905362	High SDI
Portugal	0.777927627	High-middle SDI
Spain	0.824616837	High SDI
Sweden	0.883490275	High SDI
Stockholm	0.914447593	High SDI
Sweden except Stockholm	0.872833379	High SDI
Switzerland	0.888752501	High SDI
United Kingdom	0.843093074	High SDI
England	0.848869853	High SDI
East Midlands	0.83007704	High SDI
East of England	0.840300066	High SDI
Greater London	0.894369062	High SDI
North East England	0.820735615	High SDI
North West England	0.833664296	High SDI
South East England	0.856169812	High SDI
South West England	0.841270041	High SDI
West Midlands	0.829368047	High SDI
Yorkshire and the Humber	0.829690925	High SDI
Northern Ireland	0.835352065	High SDI
Scotland	0.805372811	High SDI
Wales	0.805748561	High SDI
Latin America and Caribbean	0.639865451	

Andean Latin America	0.628313955	
Bolivia	0.587409304	Low-middle SDI
Ecuador	0.635566909	Middle SDI
Peru	0.635787809	Middle SDI
Caribbean	0.637604561	
Antigua and Barbuda	0.715130979	High-middle SDI
The Bahamas	0.75556215	High-middle SDI
Barbados	0.739423177	High-middle SDI
Belize	0.602243591	Low-middle SDI
Bermuda	0.80545317	High-middle SDI
Cuba	0.687667664	Middle SDI
Dominica	0.68658657	Middle SDI
Dominican Republic	0.592640504	Low-middle SDI
Grenada	0.640418422	Middle SDI
Guyana	0.583747015	Low-middle SDI
Haiti	0.441665969	Low SDI
Jamaica	0.678532504	Middle SDI
Puerto Rico	0.812984477	High-middle SDI
Saint Lucia	0.652614198	Middle SDI
Saint Vincent and the Grenadines	0.608304473	Middle SDI
Suriname	0.64099299	Middle SDI
Trinidad and Tobago	0.698405348	Middle SDI
Virgin Islands, U.S.	0.806568682	High-middle SDI
Central Latin America	0.623192305	
Colombia	0.633692252	Middle SDI
Costa Rica	0.662129526	Middle SDI
El Salvador	0.59309467	Low-middle SDI
Guatemala	0.524214498	Low-middle SDI
Honduras	0.512339813	Low-middle SDI
Mexico	0.628360997	Middle SDI
Aguascalientes	0.659089353	Middle SDI
Baja California	0.656785464	Middle SDI
Baja California Sur	0.658976353	Middle SDI
Campeche	0.615914899	Middle SDI
Chiapas	0.53276266	Middle SDI
Chihuahua	0.638589391	Middle SDI
Coahuila	0.645326148	Middle SDI
Colima	0.65420353	Middle SDI
Durango	0.623979236	Middle SDI
Guanajuato	0.62129178	Middle SDI
Guerrero	0.562442968	Middle SDI
Hidalgo	0.587458446	Middle SDI
Jalisco	0.648991934	Middle SDI
Mexico	0.635428465	Middle SDI
Mexico City	0.715772109	Middle SDI
Michoacan de Ocampo	0.58646838	Middle SDI

Morelos	0.635471941	Middle SDI
Nayarit	0.620025881	Middle SDI
Nuevo Leon	0.677420872	Middle SDI
Oaxaca	0.560543467	Middle SDI
Puebla	0.584252823	Middle SDI
Queretaro	0.639127345	Middle SDI
Quintana Roo	0.626303085	Middle SDI
San Luis Potosi	0.620944629	Middle SDI
Sinaloa	0.648534168	Middle SDI
Sonora	0.650495685	Middle SDI
Tabasco	0.611463527	Middle SDI
Tamaulipas	0.647006129	Middle SDI
Tlaxcala	0.604441163	Middle SDI
Veracruz de Ignacio de la Llave	0.591994	Middle SDI
Yucatan	0.63033024	Middle SDI
Zacatecas	0.607654208	Middle SDI
Nicaragua	0.529616174	Low-middle SDI
Panama	0.677043867	Middle SDI
Venezuela	0.655413104	Middle SDI
Tropical Latin America	0.662126282	
Brazil	0.663312473	Middle SDI
Acre	0.601605235	Low-middle SDI
Alagoas	0.555715012	Low-middle SDI
Amapa	0.658517629	Middle SDI
Amazonas	0.629315711	Middle SDI
Bahia	0.591019766	Low-middle SDI
Ceara	0.599501511	Low-middle SDI
Distrito Federal	0.79189036	High-middle SDI
Espirito Santo	0.676646695	Middle SDI
Goias	0.650146424	Middle SDI
Maranhao	0.507040138	Low-middle SDI
Mato Grosso	0.662454796	Middle SDI
Mato Grosso do Sul	0.650210546	Middle SDI
Minas Gerais	0.660795264	Middle SDI
Para	0.578664243	Low-middle SDI
Paraiba	0.574462555	Low-middle SDI
Parana	0.682436727	Middle SDI
Pernambuco	0.593552542	Low-middle SDI
Piaui	0.551619925	Low-middle SDI
Rio de Janeiro	0.708855843	High-middle SDI
Rio Grande do Norte	0.605294307	Low-middle SDI
Rio Grande do Sul	0.6927427	Middle SDI
Rondonia	0.621702361	Middle SDI
Roraima	0.646354751	Middle SDI
Santa Catarina	0.702495682	High-middle SDI
Sao Paulo	0.7200519	High-middle SDI

Sergipe	0.615627706	Middle SDI
Tocantins	0.610879077	Middle SDI
Paraguay	0.618769591	Middle SDI
North Africa and Middle East	0.638603537	
North Africa and Middle East	0.638603537	
Afghanistan	0.290254968	Low SDI
Algeria	0.695849021	Middle SDI
Bahrain	0.712258604	High-middle SDI
Egypt	0.604307711	Low-middle SDI
Iran	0.700086759	High-middle SDI
Iraq	0.584823813	Low-middle SDI
Jordan	0.696845045	Middle SDI
Kuwait	0.785593198	High-middle SDI
Lebanon	0.729621127	High-middle SDI
Libya	0.760934217	High-middle SDI
Morocco	0.579231309	Low-middle SDI
Palestine	0.541353069	Low-middle SDI
Oman	0.743531097	High-middle SDI
Qatar	0.765715882	High-middle SDI
Saudi Arabia	0.7790137	High-middle SDI
Sudan	0.477915229	Low-middle SDI
Syria	0.611084286	Middle SDI
Tunisia	0.675428611	Middle SDI
Turkey	0.729481001	High-middle SDI
United Arab Emirates	0.794722025	High-middle SDI
Yemen	0.429504407	Low SDI
South Asia	0.533975763	
South Asia	0.533975763	
Bangladesh	0.457988721	Low SDI
Bhutan	0.569907913	Low-middle SDI
India	0.550242018	Low-middle SDI
Nepal	0.428511471	Low SDI
Pakistan	0.492158484	Low-middle SDI
Southeast Asia, East Asia, and Oceania	0.685403755	
East Asia	0.708630758	
China	0.707319288	High-middle SDI
North Korea	0.537679957	Low-middle SDI
Taiwan	0.86418562	High SDI
Oceania	0.470985744	
American Samoa	0.701859796	High-middle SDI
Federated States of Micronesia	0.575251612	Low-middle SDI
Fiji	0.641435501	Middle SDI
Guam	0.794193119	High-middle SDI
Kiribati	0.426768011	Low SDI
Marshall Islands	0.550457832	Low-middle SDI
Northern Mariana Islands	0.75781722	High-middle SDI

Papua New Guinea	0.418998443	Low SDI
Samoa	0.576375166	Low-middle SDI
Solomon Islands	0.425018528	Low SDI
Tonga	0.624951156	Middle SDI
Vanuatu	0.475309121	Low-middle SDI
Southeast Asia	0.640717246	
Cambodia	0.481619391	Low-middle SDI
Indonesia	0.647611359	Middle SDI
Aceh	0.640414411	Middle SDI
Bali	0.646777358	Middle SDI
Bangka-Belitung Islands	0.637063919	Middle SDI
Banten	0.636136405	Middle SDI
Bengkulu	0.605588458	Low-middle SDI
Gorontalo	0.556881893	Low-middle SDI
Jakarta	0.795041917	High-middle SDI
Jambi	0.640546524	Middle SDI
West Java	0.635672599	Middle SDI
Central Java	0.606724047	Middle SDI
East Java	0.64169154	Middle SDI
West Kalimantan	0.589201584	Low-middle SDI
South Kalimantan	0.623798672	Middle SDI
Central Kalimantan	0.641894718	Middle SDI
East Kalimantan	0.746595227	High-middle SDI
North Kalimantan	0.755952734	High-middle SDI
Riau Islands	0.727599596	High-middle SDI
Lampung	0.616299987	Middle SDI
Maluku	0.555610326	Low-middle SDI
North Maluku	0.546157963	Low-middle SDI
West Nusa Tenggara	0.556566054	Low-middle SDI
East Nusa Tenggara	0.518912804	Low-middle SDI
Papua	0.587862719	Low-middle SDI
West Papua	0.683007739	Middle SDI
Riau	0.713955299	High-middle SDI
West Sulawesi	0.559336878	Low-middle SDI
South Sulawesi	0.610967812	Middle SDI
Central Sulawesi	0.612199879	Middle SDI
Southeast Sulawesi	0.596388581	Low-middle SDI
North Sulawesi	0.651649236	Middle SDI
West Sumatra	0.640858055	Middle SDI
South Sumatra	0.642344679	Middle SDI
North Sumatra	0.653390877	Middle SDI
Yogyakarta	0.65012062	Middle SDI
Laos	0.518788871	Low-middle SDI
Malaysia	0.759248836	High-middle SDI
Maldives	0.655286841	Middle SDI
Mauritius	0.720190502	High-middle SDI

Myanmar	0.555817824	Low-middle SDI
Philippines	0.617174396	Middle SDI
Sri Lanka	0.679706328	Middle SDI
Seychelles	0.692334035	Middle SDI
Thailand	0.684276785	Middle SDI
Timor-Leste	0.504842989	Low-middle SDI
Vietnam	0.606829222	Middle SDI
Sub-Saharan Africa	0.445980066	
Central Sub-Saharan Africa	0.45690943	
Angola	0.460535938	Low-middle SDI
Central African Republic	0.334449009	Low SDI
Congo	0.574129526	Low-middle SDI
Democratic Republic of the Congo	0.364453165	Low SDI
Equatorial Guinea	0.62522322	Middle SDI
Gabon	0.650559028	Middle SDI
Eastern Sub-Saharan Africa	0.387060963	
Burundi	0.309705632	Low SDI
Comoros	0.434289553	Low SDI
Djibouti	0.484750347	Low-middle SDI
Eritrea	0.408790995	Low SDI
Ethiopia	0.334181415	Low SDI
Kenya	0.499471993	Low-middle SDI
Madagascar	0.330760552	Low SDI
Malawi	0.349345085	Low SDI
Mozambique	0.340470577	Low SDI
Rwanda	0.40744149	Low SDI
Somalia	0.234806633	Low SDI
South Sudan	0.274705978	Low SDI
Tanzania	0.412207128	Low SDI
Uganda	0.387738241	Low SDI
Zambia	0.472213354	Low-middle SDI
Southern Sub-Saharan Africa	0.639979771	
Botswana	0.663238118	Middle SDI
Lesotho	0.493356884	Low-middle SDI
Namibia	0.615792035	Middle SDI
South Africa	0.676542582	Middle SDI
Swaziland	0.577699713	Low-middle SDI
Zimbabwe	0.463195841	Low-middle SDI
Western Sub-Saharan Africa	0.441032713	
Benin	0.373374857	Low SDI
Burkina Faso	0.283938202	Low SDI
Cameroon	0.482039386	Low-middle SDI
Cape Verde	0.549086441	Low-middle SDI
Chad	0.252901641	Low SDI
Cote d'Ivoire	0.412139874	Low SDI
The Gambia	0.404759628	Low SDI

Ghana	0.536972566	Low-middle SDI
Guinea	0.324710505	Low SDI
Guinea-Bissau	0.348986787	Low SDI
Liberia	0.328416338	Low SDI
Mali	0.266900909	Low SDI
Mauritania	0.470565798	Low-middle SDI
Niger	0.190617687	Low SDI
Nigeria	0.49339389	Low-middle SDI
Sao Tome and Principe	0.488258275	Low-middle SDI
Senegal	0.373026564	Low SDI
Sierra Leone	0.357159036	Low SDI
Togo	0.413313302	Low SDI

INFERTILITY CASE DEFINITION AND MODELLING SUMMARY

For GBD 2017, the following case definitions were used for infertility:

1. Primary infertility is defined as a couple who have not had a livebirth, who wish a child, and have been in a union for more than five years without using contraceptives.

2. Secondary infertility is defined in a couple who wish a child and have been in a union for more than five years without using contraceptives since the last livebirth.

Estimation is completed in three steps. First, we estimate total primary (unable to have any child) and secondary (unable to have an additional child) infertility in couples. This is accomplished by first quantifying the rate of infertility among survey respondents who are married (the subset to whom such questions are directed) and then quantifying how the married population relates to the overall population. Second, we model which proportion of primary and secondary infertility is due to female and male factor, respectively, to estimate four "envelopes" of infertility: male primary infertility, male secondary infertility, female primary infertility, and female secondary infertility. Third, we execute a "causal arrtibution" process to assign cases of each envelope to likely underlying causes and assingn the remainder to idiopathic infertility (ie, unknown causes).

Input data

Our primary data sources are population surveys. The datasets were last updated for GBD 2015. Data extraction included data for women in five-year age groups between 15 and 49 from population-based surveys including the Demographic and Health Surveys (DHS), World Fertility Surveys (WFS), Reproductive Health Surveys (RHS), Family and Fertility Survey (FFS), and others (EUR, NSF, PCD, PFM). Such surveys only ask fertility-related questions to married women. Even though only women are interviewed, we treated the responses as a proxy for the infertility of couples in unions because the questions are not structured in a way that it is possible to determine which partner is the cause of the couples' inability to conceive a child.

The desire to have a child is the crucial determinant of whether a couple is labeled as infertile (ie, if no child is wanted, infertility is not present).

The combination of variables in surveys that were used to construct each of the four datasets (primary "impairment" and "exposure" and secondary "impairment" and "exposure")are illustrated in the table below. As described below, overall primary and secondary infertility are estimated by multiplying prevalence among those with the "impairment" of infertility (married women who desire a[nother] child) by the prevalence of the "exposure" (being married for 5+ years, not using contraception for 5+ years).

Model name	Infertility type	Numerator	Denominator
Primary (impairment)	Exposure to primary infertility among married women	Married 5+ years; no contraception for 5+ years prior to survey; no previous births; desires a child.	Married 5+ years

Primary (exposure)	Prevalence of exposure	Married 5+ years; no contraception for 5+ plus years prior to survey	All women
Secondary (impairment)	Exposure to secondary infertility among married women	Married 5+ years; no contraception for 5+ years prior to survey; last birth 5+ years ago; desires a child.	Married 5+ years; 1+ children
Secondary (exposure)	Prevalence of exposure to secondary infertility	married 5+ years; no contraception for 5+ years prior to survey; 1+ children	All women

The table below illustrates the extent of data coverage for the infertility envelope models for GBD 2017.

Primary infertility impairment	Prevalence
Site-years (total)	325
Number of countries with data	113
Number of GBD regions with data (out of 21 regions)	20
Number of GBD super-regions with data (out of 7 super-regions)	7
Primary infertility exposure	Prevalence
Site-years (total)	274
Number of countries with data	101
Number of GBD regions with data (out of 21 regions)	17
Number of GBD super-regions with data (out of 7 super-regions)	7
Secondary infertility impairment	Prevalence
Site-years (total)	327
Number of countries with data	112
Number of GBD regions with data (out of 21 regions)	19
Number of GBD super-regions with data (out of 7 super-regions)	7
Secondary infertility exposure	Prevalence
Site-years (total)	274
Number of countries with data	101
Number of GBD regions with data (out of 21 regions)	17
Number of GBD super-regions with data (out of 7 super-regions)	7

The second set of four datasets informed estimates of which component of primary and secondary infertility were due to female and male factors, respectively. To obtain data on the sex and cause breakdown for infertility, we systematically searched the literature in GBD 2010 using the following search string:

causes[Title/abstract] AND infertility[Title] NOT mouse NOT murine NOT rat NOT rodent

We received 626 hits from PubMed and excluded studies according to the following exclusion criteria:

1. studies not representative of the national population;
2. studies that provide no raw data,
3. studies that provide only estimates;
4. studies performed before 1970;
5. case studies or studies with sample size less than 50;
6. studies that provide no data on the sex of the partner responsible for infertility among couples.

The majority of excluded studies were excluded because of the latter criterion. In total, 15 studies were included in our analysis for the sex breakdown among infertile couples. Infertility among couples was reported as due to one of the following causes: male factor, female factor, both, or unknown. Couples with infertility due to both partners were allocated to both male factor and female factor, and couples with infertility of unknown cause were allocated to male and female factors based on the proportion observed in other couples in the study. We estimated the proportion of couples' infertility due to male factors and female factors separately in DisMod-MR 2.1. The quantity modelled was the proportion of couples' infertility due to each sex for each of primary and secondary infertility. The table below shows the dataset contents for these four models, each of which used the same set of sources.

Proportion sex-specific primary and secondary infertility	Proportion
Site-years (total)	19
Number of countries with data	15
Number of GBD regions with data (out of 21 regions)	8
Number of GBD super-regions with data (out of 7 super-regions)	6

Modelling strategy

For GBD 2017, we estimated the prevalence of primary and secondary infertility by sex and cause in three steps: 1) estimation of couples infertility [four DisMod-MR 2.1 models], 2) estimation of infertility by sex [four

DisMod-MR 2.1 models], and 3) causal attribution of infertility. We assumed zero infertility prior to age 15 or after age 50 years as fertility is not expected to be desired outside these age ranges in women; an assumption that was therefore carried over to men as well. All DisMod-MR 2.1 models were run as single parameter models. No study or country covariates were used in any models.

Estimation of couples' infertility

To estimate the prevalence of primary and secondary infertility among couples, we first run four DisModMR 2.1 models to estimate the four parameters detailed above, prevalence of primary infertility (1), prevalence of primary infertility exposure (2), prevalence of secondary infertility (3), and prevalence of secondary infertility exposure (4). For prevalence of infertility (models 1 and 3), we tried using the natural log of the age-standardised death rate (lnASDR) of sexually transmitted infections (STIs), but it was not statistically significant so we did not use it in the final model. We did not use any study- or country-level covariates for these models. Next, we estimated primary and secondary couples' infertility form DisMod-covariates for these models. Next, we estimated primary and secondary couples' infertility form DisMod-MR 2.1 models by multiplying the estimates for prevalence of infertility among exposed women by the prevalence of exposure to infertility to obtain prevalence of infertility among all women and all men.

Estimation of infertility by sex

After running the four models estimating overall infertility, described above, we ran four DisMod-MR 2.1 models to estimate the proportion of primary and secondary infertility by sex, proportion of primary female infertility, proportion of secondary female infertility, proportion of primary male infertility, and proportion of secondary male infertility. We model sex-specific infertility as a proportion. Because infertility in some couples is attributable to both partners rather than just one, the sum of the proportions due to each partner is greater than one when both partners are infertile. When the sum of the proportions is lower than one, we scale it to be equal to one through custom code. Again, we tried using lnASDR of STIs as a covariate, but it was not statistically significant so we did not use it in the final model. We did not use any study- or country-level covariates for these models. We multiplied our prevalence of primary and secondary infertility derived in step 1 by the proportion due to male and female factors to estimate primary and secondary infertility by sex.

Causal attribution

There are seven identified causes of female infertility in the GBD 2017 cause list: pelvic inflammatory disease (PID) due to chlamydia, PID due to gonorrhoea, PID due to other sexually transmitted diseases, maternal sepsis, polycystic ovarian syndrome, endometriosis, and Turner syndrome. For each of these diseases, we determined the prevalence of infertility by a literature review of the probability of becoming infertile due to that disease. For STIs, we applied a proportion with infertility derived from Westrom and colleagues1 to incident cases of PID and used DisMod-MR 2.1 to calculate corresponding prevalence for each subsequent age group through the fertile years, assuming zero remission or excess mortality. For the others, we added all the disease-specific estimates of prevalence and assigned the remaining proportion to categories of "female primary infertility due to other causes" and "female secondary infertility due to other causes." We assumed all infertility form Turner syndrome is primary infertility and all infertility following maternal sepsis is secondary infertility. The only recognized cause of male infertility in the GBD 2018 cause list is Klinefelter syndrome. We assigned all other male infertility to "male" infertility due to other causes.

Sequelae/disability weights

Every person with infertility was assumed to experience the health state as determined from the GBD disability weights survey. The lay descriptions of primary and secondary are listed below.

Health state name	Health state description	Disability weight
Infertility, primary	This person wants to have a child and has a fertile partner, but the couple cannot conceive.	0.008(0.003-0.015)
Infertility, secondary	This person has at least one child, and wants to have more children. The person has a fertile partner, but the couple cannot conceive.	0.005(0.002-0.011)

ESTIMATION PROCESS FOR DALYS

Computing DALYs

To estimate DALYs for GBD 2017, we started by estimating cause-specific mortality and non-fatal health

loss. For each year for which YLDs have been estimated (1990, 1995, 2000, 2007, 2010 and 2017), we compute DALYs by adding YLLs and YLDs for each age-sex-location. Uncertainty in YLLs was assumed to be independent of uncertainty in YLDs. We calculated 1,000 draws for DALYs by summing the first draw of the 1000 draws for YLLs and YLDs and then repeating for each subsequent draw. 95% uncertainty intervals (UI) were computed using the 25th and 975th ordered draw of the DALY uncertainty distribution. Please refer to the appendices of the GBD 2017 non-fatal capstone and cause of death capstone publications for information on how YLLs and YLDs were computed. We calculate DALYs as the sum of YLLs and YLDs for each cause, location, age group, sex, and year.
