# Supplementary material for: Global, regional, and national prevalence and disability-adjusted life-years for infertility in 195 countries and territories, 1990–2017: results from a global burden of disease study, 2017
Source: Aging (Albany NY). 2019 Dec 2;11(23):10952–91. doi: 10.18632/aging.102497 (PMC6932903; doi:10.18632/aging.102497)
Supplement: Supplementary Tables [file aging-11-102497-s003..pdf]

SUPPLEMENTARY TABLE

Supplementary Table 1. Trends in infertility age-standardized prevalence by sociodemographic index and region from 1990-2017.

Characteristics	female								male							
	PC[a] (%)			APC[b] (%)					PC[a] (%)			APC[b] (%)				
	Value	Rank		Value	95%CI[c]	95%CI[c]	Rank		Value	Rank		Value	95%CI[c]	95%CI[c]	Rank	
Global	14.962			0.370	0.213	0.527			8.224		↑	0.291	0.241	0.341		
Sociodemographic index																
Low	5.818	5	↑	0.274	-0.052	0.601	2	↑	9.893	2	↑	0.385	0.204	0.566	1	↑
Low-middle	9.856	2	↑	0.093	-0.221	0.408	5	↑	10.907	1	↑	0.177	-0.035	0.390	4	↑
Middle	9.529	3	↑	0.217	0.109	0.325	3	↑	4.936	3	↑	0.208	0.167	0.248	3	↑
High-Middle	6.205	4	↑	0.140	0.091	0.188	4	↑	-2.395	1	↓	0.155	0.049	0.261	5	↑
High	25.152	1	↑	0.766	0.591	0.942	1	↑	3.241	4	↑	0.216	0.156	0.277	2	↑
Region																
Central Asia	5.086	10	↑	0.170	-0.052	0.393	11	↑	-4.120	2	↓	0.174	0.062	0.287	12	↑
East Asia	5.005	11	↑	0.058	-0.010	0.126	14	↑	-2.545	5	↓	0.058	-0.033	0.150	16	↑
High-income Asia Pacific	2.924	12	↑	-0.096	-0.298	0.106	7	↓	-3.870	3	↓	0.078	-0.060	0.216	13	↑
South Asia	16.179	5	↑	0.538	0.208	0.869	8	↑	10.525	4	↑	0.313	0.120	0.506	8	↑
Southeast Asia	32.009	2	↑	0.965	0.603	1.329	4	↑	20.804	2	↑	0.660	0.412	0.909	3	↑
Central Europe	23.733	4	↑	0.591	0.420	0.763	5	↑	-4.908	1	↓	0.217	0.023	0.412	11	↑
Eastern Europe	7.320	8	↑	0.138	-0.012	0.289	12	↑	-3.430	4	↓	0.230	0.104	0.356	10	↑
Western Europe	10.458	7	↑	0.324	0.199	0.449	9	↑	5.118	8	↑	0.314	0.201	0.427	7	↑
Andean Latin America	-7.515	3	↓	2.129	0.955	3.317	1	↑	20.128	3	↑	1.558	1.203	1.913	1	↑
Central Latin America	44.729	1	↑	0.573	0.365	0.781	7	↑	28.420	1	↑	0.578	0.410	0.747	5	↑
Southern Latin America	-15.196	1	↓	-0.723	-0.880	-0.565	2	↓	0.449	11	↑	0.058	0.042	0.075	15	↑
Tropical Latin America	13.300	6	↑	1.504	0.928	2.083	2	↑	9.509	5	↑	0.926	0.654	1.199	2	↑
High income North America	6.195	9	↑	-0.730	-1.801	0.354	1	↓	0.521	10	↑	-0.347	-0.563	-0.130	1	↓
Central Sub-Saharan Africa	-2.351	5	↓	0.585	-0.012	1.187	6	↑	2.270	9	↑	0.539	0.143	0.937	6	↑
Eastern Sub-Saharan Africa	-0.756	7	↓	-0.395	-0.927	0.139	4	↓	-1.539	8	↓	-0.307	-0.608	-0.005	2	↓
Southern Sub-Saharan Africa	-0.994	6	↓	0.262	-0.013	0.538	10	↑	-0.613	9	↓	0.267	0.096	0.439	9	↑
Western Sub-Saharan Africa	1.388	13	↑	-0.662	-1.182	-0.140	3	↓	5.206	7	↑	-0.304	-0.639	0.033	3	↓
North Africa and Middle East	30.368	3	↑	1.352	1.113	1.592	3	↑	9.027	6	↑	0.601	0.478	0.723	4	↑
Oceania	-11.370	2	↓	-0.222	-0.361	-0.084	6	↓	0.024	12	↑	-0.003	-0.015	0.010	5	↓
Australasia	0.949	14	↑	0.080	0.009	0.151	13	↑	-2.232	7	↓	0.060	-0.033	0.154	14	↑
Caribbean	-4.943	4	↓	-0.239	-0.306	-0.171	5	↓	-2.518	6	↓	-0.111	-0.147	-0.074	4	↓

a: percent change.
b: annual percent change
c: confidence interval

Supplementary Table 2. The relative contributions of each geographical locations in trends of infertility prevalence from 1990-2017.

Characteristics	female		Male	
	Increasing trend	Decreasing trend	Increasing trend	Decreasing trend
	Contribution rate (%)	Contribution rate (%)		
Sociodemographic index				
Low	18.37		33.75	
Low-middle	6.26		15.52	
Middle	14.58		18.20	
Middle-High	9.39		13.60	
High	51.41		18.93	
Region	-			
Central Asia	1.84		2.63	
Eastern Asia	0.63		0.88	
High-income Asia Pacific		3.13	1.17	
South Asia	5.80		4.72	
Southeast Asia	10.41		9.95	
Central Europe	6.38		3.28	
Eastern Europe	1.49		3.47	
Western Europe	3.49		4.74	
Andean Latin America	22.97		23.48	
Central Latin America	6.18		8.72	
Southern Latin America		23.56	0.88	
Tropical Latin America	16.22		13.96	
North America		23.79		32.40
Central Sub-Saharan Africa	6.31		8.13	
Eastern Sub-Saharan Africa		12.89		28.67
Southern Sub-Saharan Africa	2.83		4.03	
Western Sub-Saharan Africa		21.60		28.37
North Africa and Middle East	14.59		9.05	
Oceania		7.25		0.24
Australasia	0.86		0.91	
Caribbean		7.78		10.33

Please browse Full Text version to see the data of Supplementary Table 3.

Supplementary Table 3. Trends in infertility age-standardized prevalence rate of 195 countries and territories from 1990-2017.

Supplementary Table 4. Trends in infertility age-standardized DALYs by sociodemographic index and region from 1990-2017.

Characteristics	female								male							
	PC[a] (%)			APC[b] (%)					PC[a] (%)			APC[b] (%)				
	Value	Rank		Value	95%CI[c]	95%CI[c]	Rank		Value	Rank		Value	95%CI[c]	95%CI[c]	Rank	
Global	15.834			0.396	0.239	0.552			8.843		↑	0.293	0.237	0.349		
Sociodemographic index																
Low	6.196	5	↑	0.279	-0.040	0.598	2	↑	9.205	2	↑	0.345	0.169	0.522	1	↑
Low-middle	10.208	3	↑	0.117	-0.190	0.424	5	↑	10.574	1	↑	0.172	-0.036	0.381	4	↑
Middle	10.591	2	↑	0.245	0.139	0.350	3	↑	6.118	3	↑	0.226	0.192	0.260	2	↑
High-Middle	6.919	4	↑	0.167	0.119	0.215	4	↑	-0.970	1	↓	0.175	0.084	0.266	3	↑
High	23.560	1	↑	0.714	0.542	0.888	1	↑	2.991	4	↑	0.166	0.120	0.211	5	↑
Region																
Central Asia	4.768	11	↑	0.165	-0.048	0.378	11	↑	-3.686	3	↓	0.153	0.053	0.253	12	↑
East Asia	5.714	9	↑	0.076	0.011	0.141	13	↑	-1.584	6	↓	0.063	-0.023	0.149	13	↑
High-income Asia Pacific	2.583	12	↑	-0.103	-0.300	0.094	7	↓	-3.910	2	↓	0.050	-0.070	0.170	15	↑
South Asia	16.036	5	↑	0.512	0.195	0.829	8	↑	10.057	4	↑	0.275	0.087	0.463	8	↑
Southeast Asia	29.602	2	↑	0.873	0.540	1.208	4	↑	17.639	3	↑	0.524	0.308	0.742	6	↑
Central Europe	22.728	4	↑	0.566	0.402	0.729	7	↑	-4.065	1	↓	0.201	0.029	0.374	11	↑
Eastern Europe	6.717	8	↑	0.134	-0.008	0.276	12	↑	-2.646	5	↓	0.205	0.100	0.310	10	↑
Western Europe	10.123	7	↑	0.322	0.201	0.443	9	↑	5.270	7	↑	0.298	0.198	0.398	7	↑
Andean Latin America	-5.284	3	↓	2.200	1.039	3.375	1	↑	19.162	2	↑	1.436	1.116	1.757	1	↑
Central Latin America	44.105	1	↑	0.574	0.369	0.779	6	↑	26.378	1	↑	0.543	0.388	0.697	3	↑
Southern Latin America	-14.713	1	↓	-0.694	-0.844	-0.543	2	↓	0.464	11	↑	0.048	0.035	0.062	16	↑
Tropical Latin America	11.425	6	↑	1.487	0.896	2.081	2	↑	7.382	6	↑	0.871	0.596	1.147	2	↑
High income North America	5.578	10	↑	-0.751	-1.814	0.324	1	↓	0.597	10	↑	-0.373	-0.613	-0.132	1	↓
Central Sub-Saharan Africa	-1.438	5	↓	0.619	0.027	1.213	5	↑	2.349	9	↑	0.542	0.158	0.927	4	↑
Eastern Sub-Saharan Africa	0.038	15	↑	-0.357	-0.875	0.164	4	↓	-0.827	8	↓	-0.281	-0.578	0.017	3	↓
Southern Sub-Saharan Africa	-1.425	6	↓	0.260	-0.013	0.535	10	↑	-0.744	9	↓	0.268	0.098	0.439	9	↑
Western Sub-Saharan Africa	1.834	13	↑	-0.635	-1.160	-0.107	3	↓	4.740	8	↑	-0.311	-0.652	0.031	2	↓
North Africa and Middle East	27.877	3	↑	1.273	1.034	1.512	3	↑	7.706	5	↑	0.532	0.414	0.649	5	↑
Oceania	-10.388	2	↓	-0.207	-0.331	-0.083	6	↓	-0.052	10	↓	-0.008	-0.019	0.003	5	↓
Australasia	1.019	14	↑	0.067	0.002	0.132	14	↑	-1.536	7	↓	0.057	-0.017	0.131	14	↑
Caribbean	-4.886	4	↓	-0.225	-0.287	-0.163	5	↓	-2.901	4	↓	-0.111	-0.145	-0.076	4	↓

a: percent change.
b: annual percent change
c: confidence interval

Supplementary Table 5: The relative contributions of each geographical locations in trends of infertility DALYs from 1990-2017.

Characteristics	female		Male	
	Increasing trend	Decreasing trend	Increasing trend	Decreasing trend
	Contribution rate (%)	Contribution rate (%)		
Sociodemographic index				
Low	18.31		31.83	
Low-middle	7.68		15.89	
Middle	16.08		20.85	
Middle-High	10.99		16.16	
High	46.95		15.27	
Region				
Central Asia	1.81		2.52	
Eastern Asia	0.84		1.04	
High-income Asia Pacific		3.47	0.82	
South Asia	5.61		4.53	
Southeast Asia	9.57		8.64	
Central Europe	6.20		3.32	
Eastern Europe	1.47		3.38	
Western Europe	3.53		4.91	
Andean Latin America	24.11		23.67	
Central Latin America	6.29		8.94	
Southern Latin America		23.34	0.80	
Tropical Latin America	16.29		14.36	
North America		25.27		34.42
Central Sub-Saharan Africa	6.78		8.93	
Eastern Sub-Saharan Africa		12.01		25.92
Southern Sub-Saharan Africa	2.85		4.42	
Western Sub-Saharan Africa		21.36		28.72
North Africa and Middle East	13.94		8.76	
Oceania		6.98		0.75
Australasia	0.73		0.94	
Caribbean		7.57		10.20

Please browse Full Text version to see the data of Supplementary Table 6.

Supplementary Table 6. Trends in infertility age-standardized DALYs of 195 countries and territories from 1990-2017.
