# Supplementary material for: Global, regional, and national prevalence and disability-adjusted life-years for infertility in 195 countries and territories, 1990–2017: results from a global burden of disease study, 2017
Source: Aging (Albany NY). 2019 Dec 2;11(23):10952–91. doi: 10.18632/aging.102497 (PMC6932903; doi:10.18632/aging.102497)
Supplement: Supplementary Table 3 [file aging-11-102497-s001..docx]

# Supplementary Table 3. Trends in infertility age-standardized prevalence rate of 195 countries and territories from 1990-2017.

|  | **female** | | | | | | | | **male** | | | | | | | |
| --- | --- | --- | --- | --- | --- | --- | --- | --- | --- | --- | --- | --- | --- | --- | --- | --- |
| **Countries and territories** | **PC^a^** | | | **APC^b^** | | | | | **PC^a^** | | | **APC^b^** | | | | |
|  | **Value** | **rank** | | **Value** | **95%CI^c^** | **95%CI^c^** | **rank** | | **Value** | **rank** | | **Value** | **95%CI^c^** | **95%CI^c^** | **rank** | |
| Afghanistan | -0.930 | 80 | ↓ | -0.107 | -0.143 | -0.070 | 81 | ↓ | 0.842 | 75 | **↑** | 0.035 | 0.027 | 0.044 | 116 | **↑** |
| Albania | 10.378 | 57 | ↑ | -0.675 | -1.651 | 0.312 | 29 | ↓ | -6.828 | 27 | **↓** | -0.262 | -0.495 | -0.028 | 25 | **↓** |
| Algeria | 56.010 | 9 | ↑ | 1.774 | 1.601 | 1.946 | 9 | ↑ | 12.167 | 20 | **↓** | 0.705 | 0.557 | 0.853 | 19 | **↑** |
| American Samoa | -11.167 | 32 | ↓ | -0.270 | -0.357 | -0.182 | 52 | ↓ | 0.204 | 97 | **↓** | 0.005 | -0.006 | 0.016 | 132 | **↑** |
| Andorra | 3.365 | 92 | ↑ | 0.015 | -0.052 | 0.083 | 86 | ↑ | -2.463 | 52 | **↓** | 0.000 | -0.053 | 0.053 | 135 | **↑** |
| Angola | -4.230 | 55 | ↓ | -0.101 | -0.154 | -0.048 | 83 | ↓ | 2.472 | 66 | **↓** | 0.057 | 0.024 | 0.091 | 109 | **↑** |
| Antigua and Barbuda | 1.556 | 104 | ↑ | -0.080 | -0.132 | -0.027 | 87 | ↓ | 1.970 | 70 | **↑** | 0.143 | 0.110 | 0.176 | 71 | **↑** |
| Argentina | -11.628 | 30 | ↓ | -0.671 | -0.901 | -0.440 | 30 | ↓ | 0.530 | 84 | **↑** | 0.067 | 0.049 | 0.085 | 99 | **↑** |
| Armenia | 6.960 | 66 | ↑ | 0.018 | -0.919 | 0.963 | 85 | ↑ | -4.040 | 35 | **↓** | 0.162 | -0.103 | 0.428 | 67 | **↑** |
| Australia | -2.709 | 68 | ↓ | -0.153 | -0.228 | -0.078 | 73 | ↓ | -3.024 | 46 | **↑** | 0.018 | -0.074 | 0.109 | 123 | **↑** |
| Austria | 3.272 | 93 | ↑ | 0.008 | -0.082 | 0.099 | 88 | ↑ | -2.347 | 57 | **↑** | 0.034 | -0.043 | 0.110 | 117 | **↑** |
| Azerbaijan | 5.789 | 77 | ↑ | -0.990 | -1.749 | -0.225 | 17 | ↓ | -2.373 | 56 | **↑** | -0.449 | -0.678 | -0.220 | 17 | **↓** |
| Bahrain | 7.315 | 63 | ↑ | 0.099 | -0.049 | 0.247 | 72 | ↑ | -3.372 | 40 | **↑** | -0.016 | -0.135 | 0.104 | 54 | **↓** |
| Bangladesh | 0.755 | 111 | ↑ | 1.148 | 0.510 | 1.790 | 15 | ↑ | 7.730 | 38 | **↓** | 0.720 | 0.446 | 0.995 | 16 | **↑** |
| Barbados | 2.284 | 98 | ↑ | -0.033 | -0.076 | 0.010 | 97 | ↓ | 0.618 | 79 | **↓** | 0.059 | 0.039 | 0.079 | 107 | **↑** |
| Belarus | 7.192 | 64 | ↑ | -0.179 | -0.359 | 0.001 | 69 | ↓ | -4.857 | 31 | **↑** | 0.224 | 0.068 | 0.380 | 57 | **↑** |
| Belgium | 18.253 | 35 | ↑ | 0.584 | 0.411 | 0.757 | 39 | ↑ | 8.872 | 32 | **↑** | 0.494 | 0.312 | 0.676 | 35 | **↑** |
| Belize | 19.594 | 33 | ↑ | 0.527 | 0.311 | 0.744 | 42 | ↑ | 11.102 | 22 | **↓** | 0.396 | 0.333 | 0.460 | 43 | **↑** |
| Benin | 4.306 | 85 | ↑ | -0.269 | -1.100 | 0.569 | 54 | ↓ | -1.057 | 74 | **↓** | -0.081 | -0.437 | 0.277 | 45 | **↓** |
| Bermuda | 3.119 | 96 | ↑ | -0.022 | -0.071 | 0.027 | 103 | ↓ | 0.088 | 100 | **↓** | 0.069 | 0.020 | 0.118 | 98 | **↑** |
| Bhutan | -12.465 | 26 | ↓ | -0.478 | -0.507 | -0.449 | 35 | ↓ | 0.455 | 86 | **↓** | 0.012 | 0.006 | 0.017 | 128 | **↑** |
| Bolivia | -41.587 | 4 | ↓ | -1.496 | -2.727 | -0.249 | 13 | ↓ | 4.308 | 45 | **↓** | 0.315 | -0.123 | 0.755 | 50 | **↑** |
| Bosnia and Herzegovina | 11.609 | 53 | ↑ | -0.091 | -0.339 | 0.158 | 86 | ↓ | -8.482 | 25 | **↑** | 0.158 | -0.051 | 0.367 | 68 | **↑** |
| Botswana | 21.980 | 27 | ↑ | 0.087 | -0.081 | 0.257 | 75 | ↑ | 13.482 | 17 | **↑** | 0.206 | 0.119 | 0.293 | 61 | **↑** |
| Brazil | 11.850 | 51 | ↑ | 1.504 | 0.882 | 2.130 | 11 | ↑ | 8.384 | 35 | **↓** | 0.913 | 0.626 | 1.200 | 11 | **↑** |
| Brunei | -2.261 | 69 | ↓ | -0.162 | -0.310 | -0.015 | 72 | ↓ | -0.750 | 80 | **↑** | 0.054 | -0.024 | 0.133 | 110 | **↑** |
| Bulgaria | 14.407 | 43 | ↑ | 0.154 | -0.023 | 0.331 | 64 | ↑ | -10.509 | 16 | **↑** | -0.086 | -0.234 | 0.061 | 44 | **↓** |
| Burkina Faso | -3.947 | 58 | ↓ | -1.104 | -1.963 | -0.236 | 14 | ↓ | -1.330 | 67 | **↑** | -0.541 | -1.005 | -0.075 | 15 | **↓** |
| Burundi | -39.431 | 6 | ↓ | -3.112 | -5.045 | -1.141 | 3 | ↓ | -18.514 | 6 | **↑** | -1.177 | -1.777 | -0.572 | 5 | **↓** |
| Cambodia | -17.274 | 14 | ↓ | -2.241 | -2.811 | -1.667 | 6 | ↓ | 0.071 | 101 | **↓** | -0.874 | -1.341 | -0.405 | 10 | **↓** |
| Cameroon | -3.389 | 62 | ↓ | -0.855 | -1.847 | 0.147 | 22 | ↓ | 8.508 | 34 | **↓** | -0.190 | -0.948 | 0.574 | 30 | **↓** |
| Canada | 3.723 | 89 | ↑ | 0.006 | -0.109 | 0.122 | 89 | ↑ | -1.317 | 68 | **↑** | 0.023 | -0.040 | 0.087 | 119 | **↑** |
| Cape Verde | 2.019 | 100 | ↑ | -0.135 | -0.260 | -0.010 | 76 | ↓ | -1.356 | 66 | **↑** | 0.116 | -0.001 | 0.233 | 81 | **↑** |
| Central African Republic | 11.031 | 55 | ↑ | 0.528 | 0.267 | 0.790 | 41 | ↑ | 16.716 | 15 | **↓** | 0.561 | 0.418 | 0.704 | 30 | **↑** |
| Chad | -11.668 | 29 | ↓ | 0.379 | 0.017 | 0.743 | 51 | ↑ | 2.538 | 64 | **↓** | 0.616 | 0.317 | 0.915 | 25 | **↑** |
| Chile | -21.344 | 12 | ↓ | -0.813 | -0.893 | -0.732 | 24 | ↓ | 0.272 | 94 | **↓** | 0.043 | 0.027 | 0.058 | 112 | **↑** |
| China | 5.172 | 81 | ↑ | 0.062 | -0.007 | 0.131 | 80 | ↑ | -2.515 | 51 | **↓** | 0.060 | -0.032 | 0.153 | 104 | **↑** |
| Colombia | 69.149 | 5 | ↑ | 1.754 | 1.139 | 2.373 | 10 | ↑ | 35.238 | 4 | **↓** | 1.080 | 0.880 | 1.279 | 5 | **↑** |
| Comoros | -8.050 | 40 | ↓ | 0.475 | 0.094 | 0.858 | 46 | ↑ | -1.657 | 63 | **↑** | 0.781 | 0.418 | 1.145 | 14 | **↑** |
| Congo | 1.727 | 103 | ↑ | -0.337 | -0.718 | 0.045 | 46 | ↓ | -0.022 | 90 | **↑** | -0.167 | -0.340 | 0.007 | 33 | **↓** |
| Costa Rica | 0.539 | 112 | ↑ | -0.031 | -0.066 | 0.004 | 98 | ↓ | -1.228 | 69 | **↓** | 0.062 | 0.012 | 0.113 | 101 | **↑** |
| Cote d'Ivoire | 52.943 | 10 | ↑ | 0.911 | 0.276 | 1.550 | 25 | ↑ | 37.316 | 2 | **↑** | 0.762 | 0.317 | 1.209 | 15 | **↑** |
| Croatia | 15.480 | 39 | ↑ | 0.097 | -0.164 | 0.360 | 73 | ↑ | -13.743 | 8 | **↑** | -0.089 | -0.299 | 0.121 | 41 | **↓** |
| Cuba | 3.857 | 87 | ↑ | 0.050 | 0.012 | 0.087 | 81 | ↑ | -3.125 | 44 | **↑** | -0.025 | -0.059 | 0.009 | 53 | **↓** |
| Cyprus | 1.457 | 106 | ↑ | -0.075 | -0.135 | -0.016 | 88 | ↓ | 0.354 | 90 | **↑** | 0.074 | 0.037 | 0.111 | 96 | **↑** |
| Czech Republic | 12.279 | 50 | ↑ | 0.587 | 0.329 | 0.847 | 38 | ↑ | -11.203 | 14 | **↓** | 0.097 | -0.129 | 0.324 | 88 | **↑** |
| Democratic Republic of the Congo | -5.365 | 52 | ↓ | 0.882 | -0.325 | 2.104 | 29 | ↑ | 2.379 | 67 | **↓** | 0.863 | 0.137 | 1.595 | 12 | **↑** |
| Denmark | 14.863 | 41 | ↑ | 0.376 | 0.178 | 0.575 | 52 | ↑ | 9.405 | 25 | **↑** | 0.444 | 0.302 | 0.586 | 36 | **↑** |
| Djibouti | 0.436 | 113 | ↑ | 0.277 | -0.039 | 0.594 | 57 | ↑ | -0.775 | 79 | **↑** | 0.216 | -0.042 | 0.475 | 58 | **↑** |
| Dominica | -1.258 | 79 | ↓ | -0.130 | -0.164 | -0.097 | 77 | ↓ | 3.413 | 53 | **↓** | 0.113 | 0.104 | 0.123 | 83 | **↑** |
| Dominican Republic | 6.137 | 76 | ↑ | -0.690 | -1.261 | -0.117 | 28 | ↓ | 8.092 | 37 | **↓** | -0.073 | -0.323 | 0.177 | 47 | **↓** |
| Ecuador | -13.683 | 21 | ↓ | 2.620 | 1.214 | 4.044 | 5 | ↑ | 7.398 | 39 | **↓** | 1.029 | 0.672 | 1.388 | 7 | **↑** |
| Egypt | 14.171 | 45 | ↑ | 1.230 | 0.803 | 1.659 | 12 | ↑ | -3.562 | 38 | **↓** | 0.323 | 0.151 | 0.495 | 49 | **↑** |
| El Salvador | -10.157 | 35 | ↓ | 0.411 | -0.757 | 1.594 | 50 | ↑ | -8.635 | 23 | **↓** | 0.184 | -0.198 | 0.567 | 65 | **↑** |
| Equatorial Guinea | -7.333 | 45 | ↓ | -0.393 | -0.483 | -0.303 | 42 | ↓ | 2.862 | 61 | **↑** | 0.199 | 0.133 | 0.265 | 63 | **↑** |
| Eritrea | 51.685 | 11 | ↑ | 1.845 | 1.586 | 2.106 | 8 | ↑ | 28.633 | 8 | **↑** | 1.038 | 0.810 | 1.267 | 6 | **↑** |
| Estonia | 3.212 | 94 | ↑ | -0.268 | -0.492 | -0.044 | 55 | ↓ | -2.890 | 47 | **↓** | 0.265 | 0.075 | 0.456 | 56 | **↑** |
| Ethiopia | 6.349 | 74 | ↑ | -0.027 | -0.478 | 0.427 | 100 | ↓ | -1.162 | 71 | **↑** | -0.117 | -0.329 | 0.094 | 39 | **↓** |
| Federated States of Micronesia | -12.573 | 24 | ↓ | -0.344 | -0.420 | -0.268 | 45 | ↓ | 0.033 | 103 | **↑** | -0.008 | -0.021 | 0.005 | 55 | **↓** |
| Fiji | -2.037 | 73 | ↓ | -0.025 | -0.046 | -0.004 | 101 | ↓ | -0.068 | 89 | **↑** | 0.000 | -0.012 | 0.011 | 136 | **↑** |
| Finland | 39.251 | 16 | ↑ | 0.455 | 0.216 | 0.695 | 48 | ↑ | 11.990 | 21 | **↑** | 0.308 | 0.124 | 0.491 | 51 | **↑** |
| France | 14.736 | 42 | ↑ | 0.520 | 0.367 | 0.674 | 43 | ↑ | 7.023 | 40 | **↓** | 0.380 | 0.267 | 0.493 | 46 | **↑** |
| Gabon | -13.046 | 23 | ↓ | -0.331 | -0.424 | -0.238 | 47 | ↓ | -10.433 | 17 | **↓** | -0.122 | -0.204 | -0.039 | 38 | **↓** |
| Georgia | 6.612 | 71 | ↑ | -0.011 | -0.146 | 0.124 | 105 | ↓ | -4.593 | 33 | **↑** | 0.106 | -0.005 | 0.217 | 85 | **↑** |
| Germany | 18.082 | 37 | ↑ | 0.543 | 0.340 | 0.748 | 40 | ↑ | 6.824 | 41 | **↑** | 0.413 | 0.258 | 0.568 | 42 | **↑** |
| Ghana | 48.733 | 12 | ↑ | 0.839 | 0.427 | 1.254 | 34 | ↑ | 25.666 | 12 | **↓** | 0.600 | 0.456 | 0.743 | 27 | **↑** |
| Greece | 3.787 | 88 | ↑ | 0.021 | -0.059 | 0.100 | 84 | ↑ | -2.422 | 54 | **↓** | 0.001 | -0.066 | 0.067 | 134 | **↑** |
| Greenland | -11.782 | 28 | ↓ | -0.572 | -0.741 | -0.403 | 32 | ↓ | 1.052 | 74 | **↓** | 0.060 | 0.050 | 0.069 | 106 | **↑** |
| Grenada | -3.451 | 61 | ↓ | -0.193 | -0.224 | -0.162 | 66 | ↓ | 3.819 | 49 | **↓** | 0.115 | 0.102 | 0.127 | 82 | **↑** |
| Guam | -5.429 | 51 | ↓ | -0.014 | -0.085 | 0.057 | 104 | ↓ | 0.091 | 99 | **↓** | -0.006 | -0.024 | 0.012 | 57 | **↓** |
| Guatemala | 6.551 | 72 | ↑ | 0.976 | 0.638 | 1.314 | 22 | ↑ | 13.229 | 18 | **↑** | 0.940 | 0.727 | 1.153 | 8 | **↑** |
| Guinea | -25.235 | 10 | ↓ | -0.668 | -0.866 | -0.470 | 31 | ↓ | -12.916 | 11 | **↑** | -0.238 | -0.404 | -0.071 | 27 | **↓** |
| Guinea-Bissau | -14.912 | 17 | ↓ | -0.439 | -0.578 | -0.300 | 40 | ↓ | 2.154 | 68 | **↓** | 0.039 | 0.000 | 0.077 | 115 | **↑** |
| Guyana | -3.603 | 60 | ↓ | -0.869 | -1.233 | -0.505 | 21 | ↓ | 4.010 | 46 | **↑** | -0.327 | -0.577 | -0.076 | 22 | **↓** |
| Haiti | -23.394 | 11 | ↓ | -0.489 | -0.750 | -0.228 | 34 | ↓ | -0.490 | 82 | **↑** | 0.279 | 0.079 | 0.479 | 55 | **↑** |
| Honduras | -2.250 | 70 | ↓ | 0.079 | -1.098 | 1.269 | 78 | ↑ | 2.749 | 63 | **↑** | 0.387 | -0.300 | 1.078 | 44 | **↑** |
| Hungary | 13.269 | 47 | ↑ | 0.173 | -0.040 | 0.386 | 63 | ↑ | -9.240 | 22 | **↑** | -0.102 | -0.264 | 0.062 | 40 | **↓** |
| Iceland | 0.802 | 110 | ↑ | -0.108 | -0.182 | -0.033 | 80 | ↓ | -0.843 | 77 | **↓** | 0.062 | -0.003 | 0.126 | 102 | **↑** |
| India | 29.267 | 20 | ↑ | 0.876 | 0.517 | 1.237 | 30 | ↑ | 14.162 | 16 | **↓** | 0.420 | 0.231 | 0.610 | 40 | **↑** |
| Indonesia | 62.290 | 8 | ↑ | 1.876 | 1.135 | 2.622 | 7 | ↑ | 36.642 | 3 | **↑** | 1.092 | 0.590 | 1.596 | 4 | **↑** |
| Iran | 6.645 | 70 | ↑ | -0.251 | -0.653 | 0.152 | 60 | ↓ | -1.134 | 72 | **↑** | -0.138 | -0.272 | -0.003 | 35 | **↓** |
| Iraq | 5.089 | 82 | ↑ | 0.094 | 0.007 | 0.181 | 74 | ↑ | -1.207 | 70 | **↓** | 0.020 | -0.029 | 0.069 | 121 | **↑** |
| Ireland | 3.971 | 86 | ↑ | -0.003 | -0.088 | 0.081 | 106 | ↓ | -2.400 | 55 | **↓** | 0.042 | -0.036 | 0.120 | 114 | **↑** |
| Israel | -2.071 | 72 | ↓ | -0.172 | -0.284 | -0.060 | 70 | ↓ | 0.705 | 77 | **↓** | 0.065 | 0.020 | 0.111 | 100 | **↑** |
| Italy | 17.462 | 38 | ↑ | 0.257 | -0.259 | 0.775 | 59 | ↑ | 9.326 | 26 | **↓** | 0.371 | 0.062 | 0.681 | 47 | **↑** |
| Jamaica | -1.977 | 74 | ↓ | -0.138 | -0.166 | -0.110 | 75 | ↓ | 3.188 | 58 | **↓** | 0.076 | 0.059 | 0.093 | 95 | **↑** |
| Japan | 9.850 | 59 | ↑ | 0.015 | -0.206 | 0.237 | 87 | ↑ | -3.366 | 41 | **↑** | 0.120 | -0.037 | 0.278 | 78 | **↑** |
| Jordan | 68.169 | 6 | ↑ | 1.152 | 0.670 | 1.635 | 14 | ↑ | 8.310 | 36 | **↑** | 0.201 | -0.091 | 0.495 | 62 | **↑** |
| Kazakhstan | 13.945 | 46 | ↑ | 0.946 | 0.634 | 1.258 | 24 | ↑ | -2.339 | 58 | **↓** | 0.576 | 0.325 | 0.828 | 28 | **↑** |
| Kenya | 86.021 | 3 | ↑ | 1.909 | 0.935 | 2.893 | 6 | ↑ | 26.843 | 9 | **↑** | 0.613 | 0.218 | 1.009 | 26 | **↑** |
| Kiribati | -8.013 | 42 | ↓ | -0.242 | -0.280 | -0.203 | 63 | ↓ | 0.315 | 92 | **↑** | 0.017 | 0.002 | 0.031 | 124 | **↑** |
| Kuwait | 7.168 | 65 | ↑ | 0.152 | 0.078 | 0.226 | 65 | ↑ | -2.668 | 50 | **↑** | -0.044 | -0.077 | -0.011 | 50 | **↓** |
| Kyrgyzstan | -1.599 | 76 | ↓ | 0.142 | -0.098 | 0.384 | 69 | ↑ | -9.932 | 20 | **↑** | 0.076 | -0.106 | 0.259 | 94 | **↑** |
| Laos | -8.036 | 41 | ↓ | -0.182 | -0.303 | -0.060 | 68 | ↓ | 0.449 | 87 | **↓** | 0.015 | 0.008 | 0.022 | 125 | **↑** |
| Latvia | 22.659 | 26 | ↑ | 0.621 | 0.401 | 0.842 | 37 | ↑ | 8.939 | 30 | **↓** | 0.940 | 0.685 | 1.195 | 9 | **↑** |
| Lebanon | 6.914 | 68 | ↑ | 1.138 | 0.801 | 1.477 | 16 | ↑ | -3.155 | 43 | **↑** | 0.662 | 0.387 | 0.938 | 23 | **↑** |
| Lesotho | -3.113 | 63 | ↓ | -1.595 | -2.738 | -0.438 | 12 | ↓ | -0.084 | 88 | **↑** | -0.843 | -1.563 | -0.118 | 12 | **↓** |
| Liberia | -26.504 | 9 | ↓ | -1.700 | -2.011 | -1.388 | 11 | ↓ | -12.322 | 12 | **↓** | -0.886 | -1.138 | -0.633 | 9 | **↓** |
| Libya | 30.256 | 19 | ↑ | 1.060 | 0.907 | 1.213 | 19 | ↑ | 3.216 | 57 | **↓** | 0.384 | 0.213 | 0.555 | 45 | **↑** |
| Lithuania | 26.747 | 22 | ↑ | 0.804 | 0.589 | 1.020 | 35 | ↑ | 2.871 | 60 | **↓** | 0.625 | 0.394 | 0.856 | 24 | **↑** |
| Luxembourg | -1.279 | 78 | ↓ | -0.105 | -0.196 | -0.014 | 82 | ↓ | 0.612 | 80 | **↓** | 0.085 | 0.042 | 0.128 | 91 | **↑** |
| Macedonia | 14.172 | 44 | ↑ | 0.112 | -0.134 | 0.359 | 71 | ↑ | -11.795 | 13 | **↓** | -0.066 | -0.265 | 0.133 | 48 | **↓** |
| Madagascar | 11.200 | 54 | ↑ | -0.697 | -1.531 | 0.145 | 27 | ↓ | 9.692 | 24 | **↑** | -0.194 | -0.624 | 0.238 | 29 | **↓** |
| Malawi | -40.040 | 5 | ↓ | -2.977 | -4.530 | -1.399 | 4 | ↓ | -29.248 | 4 | **↑** | -1.654 | -2.490 | -0.811 | 4 | **↓** |
| Malaysia | 1.171 | 107 | ↑ | -0.061 | -0.115 | -0.007 | 93 | ↓ | -1.477 | 64 | **↓** | 0.086 | 0.008 | 0.164 | 90 | **↑** |
| Maldives | -2.849 | 66 | ↓ | -0.491 | -0.836 | -0.145 | 33 | ↓ | -0.234 | 87 | **↑** | -0.089 | -0.184 | 0.006 | 42 | **↓** |
| Mali | -4.569 | 53 | ↓ | 0.144 | -0.164 | 0.454 | 67 | ↑ | 8.628 | 33 | **↑** | 0.553 | 0.317 | 0.791 | 31 | **↑** |
| Malta | 2.406 | 97 | ↑ | -0.067 | -0.152 | 0.018 | 90 | ↓ | -1.428 | 65 | **↑** | 0.046 | -0.021 | 0.112 | 111 | **↑** |
| Marshall Islands | -11.415 | 31 | ↓ | -0.260 | -0.353 | -0.167 | 58 | ↓ | 0.037 | 102 | **↑** | -0.007 | -0.023 | 0.008 | 56 | **↓** |
| Mauritania | 40.494 | 15 | ↑ | 0.886 | 0.597 | 1.176 | 28 | ↑ | 33.653 | 6 | **↓** | 0.853 | 0.675 | 1.031 | 13 | **↑** |
| Mauritius | 0.157 | 114 | ↑ | -0.066 | -0.125 | -0.006 | 91 | ↓ | -0.285 | 84 | **↓** | 0.116 | 0.049 | 0.184 | 80 | **↑** |
| Mexico | 69.177 | 4 | ↑ | 0.495 | 0.126 | 0.865 | 45 | ↑ | 40.283 | 1 | **↑** | 0.522 | 0.206 | 0.839 | 32 | **↑** |
| Moldova | 4.551 | 83 | ↑ | -0.474 | -1.033 | 0.088 | 36 | ↓ | -3.249 | 42 | **↑** | -0.158 | -0.359 | 0.043 | 34 | **↓** |
| Mongolia | 1.857 | 102 | ↑ | -0.128 | -0.233 | -0.022 | 78 | ↓ | 0.769 | 76 | **↓** | 0.125 | 0.079 | 0.171 | 76 | **↑** |
| Montenegro | 13.035 | 48 | ↑ | 0.148 | -0.030 | 0.326 | 66 | ↑ | -10.263 | 19 | **↓** | -0.060 | -0.209 | 0.089 | 49 | **↓** |
| Morocco | 42.270 | 13 | ↑ | 2.711 | 1.464 | 3.974 | 3 | ↑ | 26.286 | 11 | **↓** | 1.676 | 1.003 | 2.353 | 2 | **↑** |
| Mozambique | -6.579 | 47 | ↓ | -1.842 | -2.522 | -1.157 | 10 | ↓ | 2.838 | 62 | **↓** | -0.960 | -1.425 | -0.492 | 8 | **↓** |
| Myanmar | -8.233 | 39 | ↓ | -0.249 | -0.334 | -0.164 | 61 | ↓ | 0.555 | 81 | **↓** | 0.007 | -0.004 | 0.017 | 131 | **↑** |
| Namibia | -69.620 | 2 | ↓ | -5.943 | -6.743 | -5.136 | 2 | ↓ | -38.570 | 2 | **↑** | -2.181 | -2.518 | -1.844 | 2 | **↓** |
| Nepal | -11.116 | 33 | ↓ | 0.086 | -1.045 | 1.231 | 76 | ↑ | 0.469 | 85 | **↑** | -0.181 | -0.760 | 0.401 | 31 | **↓** |
| Netherlands | 2.135 | 99 | ↑ | -0.098 | -0.178 | -0.018 | 85 | ↓ | -0.886 | 76 | **↓** | 0.098 | 0.033 | 0.164 | 87 | **↑** |
| New Zealand | 20.851 | 31 | ↑ | 1.066 | 0.846 | 1.286 | 18 | ↑ | 3.528 | 52 | **↑** | 0.327 | 0.221 | 0.433 | 48 | **↑** |
| Nicaragua | -3.081 | 64 | ↓ | 0.971 | 0.317 | 1.629 | 23 | ↑ | 1.626 | 71 | **↑** | 0.673 | 0.385 | 0.961 | 22 | **↑** |
| Niger | -49.735 | 3 | ↓ | -2.743 | -3.038 | -2.449 | 5 | ↓ | -38.139 | 3 | **↑** | -1.750 | -1.898 | -1.601 | 3 | **↓** |
| Nigeria | 5.658 | 79 | ↑ | -0.994 | -1.769 | -0.212 | 16 | ↓ | 3.568 | 51 | **↑** | -0.839 | -1.396 | -0.280 | 13 | **↓** |
| North Korea | 0.982 | 108 | ↑ | -0.025 | -0.062 | 0.013 | 102 | ↓ | -2.210 | 61 | **↓** | -0.037 | -0.057 | -0.016 | 51 | **↓** |
| Northern Mariana Islands | -7.376 | 44 | ↓ | -0.252 | -0.280 | -0.223 | 59 | ↓ | 0.354 | 89 | **↓** | -0.005 | -0.024 | 0.013 | 58 | **↓** |
| Norway | 8.853 | 60 | ↑ | 0.366 | 0.304 | 0.428 | 54 | ↑ | 0.095 | 98 | **↑** | 0.022 | 0.004 | 0.041 | 120 | **↑** |
| Oman | 12.520 | 49 | ↑ | 0.342 | 0.172 | 0.513 | 55 | ↑ | -8.531 | 24 | **↑** | -0.244 | -0.376 | -0.113 | 26 | **↓** |
| Pakistan | -35.502 | 8 | ↓ | -1.851 | -2.230 | -1.469 | 9 | ↓ | -14.871 | 7 | **↓** | -0.844 | -1.124 | -0.562 | 11 | **↓** |
| Palestine | 6.956 | 67 | ↑ | 0.135 | 0.005 | 0.264 | 70 | ↑ | -2.828 | 48 | **↓** | 0.013 | -0.081 | 0.107 | 127 | **↑** |
| Panama | -3.623 | 59 | ↓ | -0.143 | -0.180 | -0.106 | 74 | ↓ | 3.338 | 54 | **↓** | 0.158 | 0.131 | 0.184 | 69 | **↑** |
| Papua New Guinea | -13.710 | 20 | ↓ | -0.304 | -0.466 | -0.142 | 49 | ↓ | 0.015 | 105 | **↓** | -0.004 | -0.017 | 0.010 | 59 | **↓** |
| Paraguay | 21.889 | 28 | ↑ | 0.909 | 0.363 | 1.458 | 27 | ↑ | 29.643 | 7 | **↓** | 0.916 | 0.668 | 1.164 | 10 | **↑** |
| Peru | 26.928 | 21 | ↑ | 3.597 | 2.191 | 5.023 | 2 | ↑ | 33.995 | 5 | **↑** | 2.265 | 1.733 | 2.800 | 1 | **↑** |
| Philippines | -1.938 | 75 | ↓ | -0.290 | -0.579 | 0.000 | 50 | ↓ | 9.084 | 29 | **↑** | 0.292 | 0.066 | 0.519 | 53 | **↑** |
| Poland | 36.620 | 17 | ↑ | 0.979 | 0.773 | 1.186 | 21 | ↑ | 0.281 | 93 | **↓** | 0.416 | 0.122 | 0.711 | 41 | **↑** |
| Portugal | 1.943 | 101 | ↑ | -0.073 | -0.150 | 0.004 | 89 | ↓ | -0.776 | 78 | **↑** | 0.061 | -0.006 | 0.129 | 103 | **↑** |
| Puerto Rico | 3.659 | 90 | ↑ | 0.025 | -0.015 | 0.066 | 83 | ↑ | -0.324 | 83 | **↑** | 0.019 | -0.027 | 0.065 | 122 | **↑** |
| Qatar | 10.331 | 58 | ↑ | 0.256 | 0.050 | 0.461 | 60 | ↑ | -6.076 | 30 | **↑** | -0.133 | -0.323 | 0.057 | 36 | **↓** |
| Romania | 7.331 | 62 | ↑ | -0.098 | -0.266 | 0.070 | 84 | ↓ | -4.760 | 32 | **↑** | 0.165 | 0.023 | 0.308 | 66 | **↑** |
| Russian Federation | 6.813 | 69 | ↑ | -0.033 | -0.165 | 0.099 | 96 | ↓ | -3.786 | 37 | **↓** | 0.129 | 0.013 | 0.244 | 75 | **↑** |
| Rwanda | 34.855 | 18 | ↑ | -0.456 | -1.858 | 0.966 | 38 | ↓ | 3.174 | 59 | **↓** | -0.502 | -0.880 | -0.124 | 16 | **↓** |
| Saint Lucia | -4.025 | 57 | ↓ | -0.232 | -0.269 | -0.194 | 65 | ↓ | 3.840 | 48 | **↑** | 0.131 | 0.120 | 0.142 | 74 | **↑** |
| Saint Vincent and the Grenadines | -1.486 | 77 | ↓ | -0.183 | -0.226 | -0.140 | 67 | ↓ | 3.297 | 55 | **↑** | 0.101 | 0.089 | 0.112 | 86 | **↑** |
| Samoa | -5.603 | 50 | ↓ | -0.169 | -0.189 | -0.150 | 71 | ↓ | 0.364 | 88 | **↓** | 0.012 | 0.001 | 0.022 | 129 | **↑** |
| Sao Tome and Principe | 5.727 | 78 | ↑ | -0.840 | -1.586 | -0.088 | 23 | ↓ | -4.318 | 34 | **↓** | -0.304 | -0.428 | -0.181 | 23 | **↓** |
| Saudi Arabia | 10.543 | 56 | ↑ | 0.219 | 0.046 | 0.393 | 61 | ↑ | -6.649 | 28 | **↓** | -0.088 | -0.236 | 0.061 | 43 | **↓** |
| Senegal | -11.906 | 27 | ↓ | -0.264 | -0.511 | -0.017 | 56 | ↓ | -2.316 | 59 | **↓** | 0.280 | -0.055 | 0.617 | 54 | **↑** |
| Serbia | 19.029 | 34 | ↑ | 0.284 | 0.069 | 0.499 | 56 | ↑ | -13.079 | 9 | **↓** | -0.179 | -0.342 | -0.015 | 32 | **↓** |
| Seychelles | 3.186 | 95 | ↑ | 0.086 | 0.059 | 0.113 | 77 | ↑ | -2.793 | 49 | **↑** | -0.080 | -0.114 | -0.045 | 46 | **↓** |
| Sierra Leone | -35.577 | 7 | ↓ | -1.954 | -2.304 | -1.603 | 8 | ↓ | -26.297 | 5 | **↑** | -1.146 | -1.460 | -0.831 | 6 | **↓** |
| Singapore | -10.902 | 34 | ↓ | -0.466 | -0.737 | -0.193 | 37 | ↓ | -0.574 | 81 | **↓** | 0.189 | 0.018 | 0.359 | 64 | **↑** |
| Slovakia | 18.183 | 36 | ↑ | 0.367 | 0.182 | 0.552 | 53 | ↑ | -13.058 | 10 | **↑** | -0.229 | -0.381 | -0.076 | 28 | **↓** |
| Slovenia | 95.694 | 2 | ↑ | 2.685 | 2.096 | 3.277 | 4 | ↑ | -6.454 | 29 | **↑** | 0.444 | 0.122 | 0.768 | 37 | **↑** |
| Solomon Islands | -14.493 | 18 | ↓ | -0.371 | -0.518 | -0.223 | 44 | ↓ | 0.205 | 96 | **↑** | 0.008 | -0.006 | 0.021 | 130 | **↑** |
| Somalia | -4.298 | 54 | ↓ | -0.115 | -0.149 | -0.081 | 79 | ↓ | 1.985 | 69 | **↑** | 0.057 | 0.045 | 0.070 | 108 | **↑** |
| South Africa | -0.840 | 81 | ↓ | 0.508 | 0.263 | 0.753 | 44 | ↑ | -1.086 | 73 | **↓** | 0.443 | 0.250 | 0.637 | 38 | **↑** |
| South Korea | -14.136 | 19 | ↓ | -0.447 | -0.684 | -0.211 | 39 | ↓ | 0.017 | 104 | **↓** | 0.112 | 0.036 | 0.187 | 84 | **↑** |
| South Sudan | -13.470 | 22 | ↓ | -0.438 | -0.545 | -0.331 | 41 | ↓ | 2.518 | 65 | **↑** | 0.060 | 0.033 | 0.088 | 105 | **↑** |
| Spain | 26.069 | 23 | ↑ | 0.909 | 0.752 | 1.066 | 26 | ↑ | 9.236 | 27 | **↑** | 0.495 | 0.357 | 0.633 | 34 | **↑** |
| Sri Lanka | 40.693 | 14 | ↑ | 0.463 | 0.215 | 0.712 | 47 | ↑ | 8.907 | 31 | **↓** | 0.025 | -0.152 | 0.201 | 118 | **↑** |
| Sudan | 21.784 | 29 | ↑ | 0.864 | 0.538 | 1.191 | 32 | ↑ | 18.387 | 14 | **↓** | 0.710 | 0.481 | 0.940 | 18 | **↑** |
| Suriname | 0.949 | 109 | ↑ | -0.064 | -0.101 | -0.027 | 92 | ↓ | 1.624 | 72 | **↓** | 0.071 | 0.061 | 0.080 | 97 | **↑** |
| Swaziland | -2.831 | 67 | ↓ | -0.966 | -1.508 | -0.422 | 18 | ↓ | -0.241 | 86 | **↓** | -0.337 | -0.558 | -0.116 | 21 | **↓** |
| Sweden | 3.564 | 91 | ↑ | -0.038 | -0.154 | 0.078 | 95 | ↓ | -3.844 | 36 | **↓** | 0.014 | -0.108 | 0.137 | 126 | **↑** |
| Switzerland | -4.152 | 56 | ↓ | -0.233 | -0.390 | -0.076 | 64 | ↓ | 0.536 | 83 | **↑** | 0.122 | 0.037 | 0.206 | 77 | **↑** |
| Syria | 23.138 | 25 | ↑ | 1.095 | 0.588 | 1.604 | 17 | ↑ | 5.501 | 43 | **↑** | 0.511 | 0.289 | 0.733 | 33 | **↑** |
| Taiwan | -2.859 | 65 | ↓ | -0.248 | -0.329 | -0.167 | 62 | ↓ | -2.101 | 62 | **↓** | 0.139 | 0.037 | 0.242 | 73 | **↑** |
| Tajikistan | -12.527 | 25 | ↓ | -0.907 | -1.138 | -0.676 | 19 | ↓ | -8.322 | 26 | **↑** | -0.364 | -0.436 | -0.292 | 19 | **↓** |
| Tanzania | 4.517 | 84 | ↑ | 0.737 | 0.203 | 1.274 | 36 | ↑ | 6.079 | 42 | **↑** | 0.684 | 0.281 | 1.089 | 21 | **↑** |
| Thailand | 25.737 | 24 | ↑ | 0.144 | -0.101 | 0.388 | 68 | ↑ | 13.100 | 19 | **↑** | 0.306 | 0.180 | 0.433 | 52 | **↑** |
| The Bahamas | -5.925 | 49 | ↓ | -0.269 | -0.308 | -0.230 | 53 | ↓ | 3.926 | 47 | **↑** | 0.117 | 0.101 | 0.132 | 79 | **↑** |
| The Gambia | -17.219 | 15 | ↓ | -1.025 | -1.250 | -0.800 | 15 | ↓ | -10.838 | 15 | **↓** | -0.572 | -0.710 | -0.434 | 14 | **↓** |
| Timor-Leste | -9.925 | 36 | ↓ | -0.871 | -1.115 | -0.625 | 20 | ↓ | 1.059 | 73 | **↓** | -0.362 | -0.605 | -0.119 | 20 | **↓** |
| Togo | 20.104 | 32 | ↑ | -0.289 | -0.823 | 0.247 | 51 | ↓ | 10.267 | 23 | **↑** | 0.079 | -0.192 | 0.349 | 93 | **↑** |
| Tonga | 1.491 | 105 | ↑ | 0.032 | 0.003 | 0.060 | 82 | ↑ | -0.961 | 75 | **↑** | -0.035 | -0.043 | -0.027 | 52 | **↓** |
| Trinidad and Tobago | 21.096 | 30 | ↑ | 0.206 | 0.045 | 0.368 | 62 | ↑ | 5.456 | 44 | **↓** | 0.154 | 0.037 | 0.271 | 70 | **↑** |
| Tunisia | 67.665 | 7 | ↑ | 1.172 | 0.665 | 1.682 | 13 | ↑ | 19.821 | 13 | **↓** | 0.711 | 0.439 | 0.983 | 17 | **↑** |
| Turkey | 116.852 | 1 | ↑ | 3.928 | 3.361 | 4.497 | 1 | ↑ | 26.671 | 10 | **↓** | 1.498 | 1.190 | 1.808 | 3 | **↑** |
| Turkmenistan | 6.148 | 75 | ↑ | -0.042 | -0.181 | 0.097 | 94 | ↓ | -3.106 | 45 | **↓** | 0.084 | -0.032 | 0.201 | 92 | **↑** |
| Uganda | -16.790 | 16 | ↓ | -2.000 | -3.473 | -0.504 | 7 | ↓ | -10.414 | 18 | **↓** | -1.059 | -1.743 | -0.371 | 7 | **↓** |
| Ukraine | 5.204 | 80 | ↑ | 0.870 | 0.400 | 1.343 | 31 | ↑ | -3.501 | 39 | **↑** | 0.564 | 0.298 | 0.830 | 29 | **↑** |
| United Arab Emirates | 15.441 | 40 | ↑ | 0.446 | 0.221 | 0.672 | 49 | ↑ | -9.617 | 21 | **↑** | -0.268 | -0.457 | -0.078 | 24 | **↓** |
| United Kingdom | -2.239 | 71 | ↓ | 0.066 | -0.147 | 0.280 | 79 | ↑ | -2.212 | 60 | **↓** | 0.088 | -0.014 | 0.189 | 89 | **↑** |
| United States | 6.358 | 73 | ↑ | -0.789 | -1.957 | 0.392 | 26 | ↓ | 0.672 | 78 | **↑** | -0.385 | -0.628 | -0.141 | 18 | **↓** |
| Uruguay | -20.480 | 13 | ↓ | -0.793 | -0.884 | -0.701 | 25 | ↓ | 0.323 | 91 | **↑** | 0.042 | 0.033 | 0.052 | 113 | **↑** |
| Uzbekistan | 8.350 | 61 | ↑ | 0.850 | 0.524 | 1.178 | 33 | ↑ | -2.426 | 53 | **↑** | 0.439 | 0.233 | 0.645 | 39 | **↑** |
| Vanuatu | -9.757 | 37 | ↓ | -0.264 | -0.390 | -0.137 | 57 | ↓ | 0.233 | 95 | **↑** | 0.003 | -0.012 | 0.019 | 133 | **↑** |
| Venezuela | -7.884 | 43 | ↓ | -0.331 | -0.380 | -0.282 | 48 | ↓ | 3.269 | 56 | **↓** | 0.213 | 0.175 | 0.251 | 59 | **↑** |
| Vietnam | -9.016 | 38 | ↓ | 1.050 | 0.437 | 1.666 | 20 | ↑ | 0.547 | 82 | **↓** | 0.688 | 0.451 | 0.925 | 20 | **↑** |
| Virgin Islands, U.S. | -6.762 | 46 | ↓ | -0.390 | -0.457 | -0.324 | 43 | ↓ | 3.604 | 50 | **↑** | 0.141 | 0.127 | 0.155 | 72 | **↑** |
| Yemen | -6.418 | 48 | ↓ | 0.257 | -0.298 | 0.816 | 58 | ↑ | -0.246 | 85 | **↑** | 0.208 | -0.099 | 0.515 | 60 | **↑** |
| Zambia | -72.749 | 1 | ↓ | -5.954 | -6.628 | -5.275 | 1 | ↓ | -48.244 | 1 | **↓** | -2.900 | -3.255 | -2.544 | 1 | **↓** |
| Zimbabwe | 11.630 | 52 | ↑ | -0.030 | -1.097 | 1.048 | 99 | ↓ | 9.201 | 28 | **↓** | -0.127 | -0.669 | 0.417 | 37 | **↓** |

a: percent change.

b: annual percent change

c: confidence interval
