# Supplementary material for: Global, regional, and national prevalence and disability-adjusted life-years for infertility in 195 countries and territories, 1990–2017: results from a global burden of disease study, 2017
Source: Aging (Albany NY). 2019 Dec 2;11(23):10952–91. doi: 10.18632/aging.102497 (PMC6932903; doi:10.18632/aging.102497)
Supplement: Supplementary Table 6 [file aging-11-102497-s002..docx]

# Supplementary Table 6. Trends in infertility age-standardized DALYs of 195 countries and territories from 1990-2017.

|  | **female** | | | | | | | | **male** | | | | | | | |
| --- | --- | --- | --- | --- | --- | --- | --- | --- | --- | --- | --- | --- | --- | --- | --- | --- |
| **Countries and territories** | **PC^a^(%)** | | | **APC^b^(%)** | | | | | **PC^a^(%)** | | | **APC^b^(%)** | | | | |
|  | **Value** | **rank** | | **Value** | **95%CI^c^** | **95%CI^c^** | **rank** | | **Value** | **rank** | | **Value** | **95%CI^c^** | **95%CI^c^** | **rank** | |
| Afghanistan | 0.412 | 113 | **↑** | -0.049 | -0.080 | -0.019 | 94 | **↓** | 2.411 | 68 | **↑** | 0.098 | 0.091 | 0.105 | 84 | **↑** |
| Albania | 10.070 | 55 | **↑** | -0.658 | -1.605 | 0.299 | 28 | **↓** | -5.692 | 28 | **↓** | -0.244 | -0.468 | -0.019 | 26 | **↓** |
| Algeria | 49.980 | 10 | **↑** | 1.663 | 1.522 | 1.804 | 10 | **↑** | 9.593 | 28 | **↑** | 0.618 | 0.469 | 0.767 | 23 | **↑** |
| American Samoa | -10.398 | 33 | **↓** | -0.255 | -0.334 | -0.176 | 50 | **↓** | 0.108 | 99 | **↑** | -0.002 | -0.012 | 0.008 | 61 | **↓** |
| Andorra | 3.163 | 90 | **↑** | 0.013 | -0.051 | 0.076 | 85 | **↑** | -2.020 | 56 | **↓** | 0.000 | -0.042 | 0.042 | 131 | **↑** |
| Angola | -3.363 | 58 | **↓** | -0.068 | -0.116 | -0.021 | 86 | **↓** | 2.501 | 65 | **↑** | 0.065 | 0.034 | 0.097 | 102 | **↑** |
| Antigua and Barbuda | 1.598 | 105 | **↑** | -0.073 | -0.122 | -0.023 | 85 | **↓** | 1.639 | 72 | **↑** | 0.119 | 0.090 | 0.148 | 72 | **↑** |
| Argentina | -11.564 | 25 | **↓** | -0.647 | -0.864 | -0.428 | 30 | **↓** | 0.499 | 83 | **↑** | 0.053 | 0.038 | 0.069 | 107 | **↑** |
| Armenia | 6.605 | 70 | **↑** | -0.006 | -0.916 | 0.913 | 105 | **↓** | -3.462 | 36 | **↓** | 0.121 | -0.138 | 0.380 | 70 | **↑** |
| Australia | -2.188 | 67 | **↓** | -0.153 | -0.228 | -0.078 | 71 | **↓** | -2.204 | 52 | **↓** | 0.018 | -0.053 | 0.090 | 125 | **↑** |
| Austria | 3.119 | 91 | **↑** | 0.074 | -0.016 | 0.165 | 79 | **↑** | -2.019 | 57 | **↓** | 0.065 | -0.001 | 0.132 | 99 | **↑** |
| Azerbaijan | 5.370 | 80 | **↑** | -0.964 | -1.691 | -0.232 | 18 | **↓** | -1.991 | 58 | **↓** | -0.430 | -0.652 | -0.208 | 17 | **↓** |
| Bahrain | 6.999 | 65 | **↑** | 0.105 | -0.026 | 0.237 | 72 | **↑** | -2.491 | 45 | **↓** | -0.006 | -0.103 | 0.090 | 59 | **↓** |
| Bangladesh | 1.348 | 106 | **↑** | 1.110 | 0.511 | 1.713 | 14 | **↑** | 6.628 | 39 | **↑** | 0.660 | 0.410 | 0.911 | 19 | **↑** |
| Barbados | 2.201 | 99 | **↑** | -0.031 | -0.071 | 0.009 | 99 | **↓** | 0.312 | 90 | **↑** | 0.051 | 0.034 | 0.067 | 110 | **↑** |
| Belarus | 6.746 | 68 | **↑** | -0.164 | -0.331 | 0.003 | 68 | **↓** | -3.910 | 32 | **↓** | 0.192 | 0.061 | 0.322 | 59 | **↑** |
| Belgium | 17.202 | 38 | **↑** | 0.551 | 0.388 | 0.714 | 41 | **↑** | 7.787 | 35 | **↑** | 0.430 | 0.275 | 0.585 | 36 | **↑** |
| Belize | 18.837 | 32 | **↑** | 0.505 | 0.302 | 0.710 | 44 | **↑** | 9.886 | 25 | **↑** | 0.353 | 0.295 | 0.410 | 44 | **↑** |
| Benin | 5.276 | 81 | **↑** | -0.212 | -1.038 | 0.621 | 61 | **↓** | -0.189 | 89 | **↓** | -0.066 | -0.430 | 0.299 | 44 | **↓** |
| Bermuda | 2.876 | 96 | **↑** | -0.019 | -0.064 | 0.026 | 102 | **↓** | 0.191 | 93 | **↑** | 0.067 | 0.025 | 0.109 | 96 | **↑** |
| Bhutan | -10.672 | 29 | **↓** | -0.409 | -0.436 | -0.383 | 38 | **↓** | 0.370 | 87 | **↑** | 0.013 | 0.007 | 0.018 | 127 | **↑** |
| Bolivia | -40.537 | 4 | **↓** | -1.427 | -2.654 | -0.184 | 13 | **↓** | 3.841 | 45 | **↑** | 0.283 | -0.133 | 0.700 | 52 | **↑** |
| Bosnia and Herzegovina | 11.221 | 53 | **↑** | -0.082 | -0.320 | 0.156 | 83 | **↓** | -7.683 | 25 | **↓** | 0.138 | -0.046 | 0.323 | 67 | **↑** |
| Botswana | 21.160 | 29 | **↑** | 0.075 | -0.090 | 0.241 | 78 | **↑** | 13.254 | 16 | **↑** | 0.203 | 0.121 | 0.285 | 58 | **↑** |
| Brazil | 9.918 | 56 | **↑** | 1.484 | 0.848 | 2.125 | 11 | **↑** | 6.327 | 41 | **↑** | 0.858 | 0.569 | 1.148 | 12 | **↑** |
| Brunei | -1.987 | 70 | **↓** | -0.160 | -0.303 | -0.016 | 69 | **↓** | -0.568 | 82 | **↓** | 0.038 | -0.027 | 0.104 | 115 | **↑** |
| Bulgaria | 14.152 | 41 | **↑** | 0.160 | -0.009 | 0.329 | 66 | **↑** | -9.116 | 19 | **↓** | -0.072 | -0.203 | 0.059 | 42 | **↓** |
| Burkina Faso | -4.033 | 53 | **↓** | -1.081 | -1.934 | -0.220 | 14 | **↓** | -2.717 | 42 | **↓** | -0.574 | -1.045 | -0.100 | 14 | **↓** |
| Burundi | -36.706 | 6 | **↓** | -2.973 | -4.848 | -1.061 | 3 | **↓** | -16.728 | 6 | **↓** | -1.099 | -1.676 | -0.518 | 6 | **↓** |
| Cambodia | -16.089 | 15 | **↓** | -2.073 | -2.617 | -1.525 | 6 | **↓** | 0.679 | 80 | **↑** | -0.698 | -1.122 | -0.271 | 12 | **↓** |
| Cameroon | -1.342 | 75 | **↓** | -0.760 | -1.755 | 0.245 | 25 | **↓** | 10.234 | 22 | **↑** | -0.129 | -0.894 | 0.643 | 35 | **↓** |
| Canada | 3.061 | 94 | **↑** | -0.005 | -0.113 | 0.103 | 106 | **↓** | -0.907 | 73 | **↓** | 0.027 | -0.022 | 0.075 | 120 | **↑** |
| Cape Verde | 2.301 | 98 | **↑** | -0.120 | -0.240 | 0.001 | 76 | **↓** | -1.104 | 69 | **↓** | 0.110 | 0.006 | 0.215 | 77 | **↑** |
| Central African Republic | 12.123 | 49 | **↑** | 0.558 | 0.306 | 0.811 | 39 | **↑** | 16.671 | 15 | **↑** | 0.567 | 0.426 | 0.707 | 28 | **↑** |
| Chad | -11.079 | 28 | **↓** | 0.408 | 0.041 | 0.777 | 51 | **↑** | 2.488 | 66 | **↑** | 0.592 | 0.296 | 0.889 | 27 | **↑** |
| Chile | -20.131 | 12 | **↓** | -0.773 | -0.848 | -0.699 | 24 | **↓** | 0.430 | 85 | **↑** | 0.039 | 0.026 | 0.053 | 114 | **↑** |
| China | 5.905 | 76 | **↑** | 0.081 | 0.015 | 0.147 | 77 | **↑** | -1.522 | 62 | **↓** | 0.065 | -0.021 | 0.152 | 100 | **↑** |
| Colombia | 68.659 | 4 | **↑** | 1.765 | 1.170 | 2.364 | 8 | **↑** | 31.637 | 4 | **↑** | 1.025 | 0.848 | 1.202 | 4 | **↑** |
| Comoros | -6.725 | 44 | **↓** | 0.610 | 0.199 | 1.022 | 38 | **↑** | -0.594 | 81 | **↓** | 0.911 | 0.515 | 1.310 | 8 | **↑** |
| Congo | 2.193 | 100 | **↑** | -0.328 | -0.708 | 0.052 | 45 | **↓** | 0.469 | 84 | **↑** | -0.172 | -0.338 | -0.005 | 31 | **↓** |
| Costa Rica | 0.821 | 111 | **↑** | -0.020 | -0.054 | 0.014 | 101 | **↓** | -0.916 | 72 | **↓** | 0.054 | 0.012 | 0.096 | 106 | **↑** |
| Cote d'Ivoire | 53.493 | 9 | **↑** | 0.925 | 0.293 | 1.562 | 25 | **↑** | 37.098 | 2 | **↑** | 0.753 | 0.312 | 1.196 | 15 | **↑** |
| Croatia | 14.989 | 40 | **↑** | 0.087 | -0.162 | 0.338 | 75 | **↑** | -12.342 | 9 | **↓** | -0.083 | -0.270 | 0.105 | 40 | **↓** |
| Cuba | 3.988 | 86 | **↑** | 0.054 | 0.018 | 0.090 | 81 | **↑** | -2.440 | 47 | **↓** | -0.015 | -0.044 | 0.014 | 55 | **↓** |
| Cyprus | 1.861 | 104 | **↑** | -0.056 | -0.110 | -0.001 | 92 | **↓** | 0.193 | 92 | **↑** | 0.063 | 0.032 | 0.094 | 105 | **↑** |
| Czech Republic | 11.633 | 50 | **↑** | 0.558 | 0.315 | 0.803 | 40 | **↑** | -10.023 | 15 | **↓** | 0.092 | -0.113 | 0.297 | 87 | **↑** |
| Democratic Republic of the Congo | -4.347 | 52 | **↓** | 0.928 | -0.268 | 2.138 | 24 | **↑** | 2.445 | 67 | **↑** | 0.866 | 0.160 | 1.578 | 11 | **↑** |
| Denmark | 15.310 | 39 | **↑** | 0.416 | 0.224 | 0.609 | 49 | **↑** | 9.039 | 29 | **↑** | 0.413 | 0.282 | 0.545 | 38 | **↑** |
| Djibouti | 0.489 | 112 | **↑** | 0.274 | -0.036 | 0.584 | 58 | **↑** | -0.752 | 76 | **↓** | 0.206 | -0.040 | 0.452 | 56 | **↑** |
| Dominica | -0.911 | 78 | **↓** | -0.113 | -0.144 | -0.081 | 77 | **↓** | 2.951 | 56 | **↑** | 0.099 | 0.090 | 0.108 | 82 | **↑** |
| Dominican Republic | 4.766 | 83 | **↑** | -0.655 | -1.198 | -0.109 | 29 | **↓** | 5.824 | 42 | **↑** | -0.062 | -0.290 | 0.167 | 46 | **↓** |
| Ecuador | -11.998 | 23 | **↓** | 2.664 | 1.276 | 4.071 | 4 | **↑** | 8.199 | 34 | **↑** | 0.953 | 0.630 | 1.278 | 6 | **↑** |
| Egypt | 8.364 | 60 | **↑** | 0.978 | 0.591 | 1.366 | 21 | **↑** | -7.759 | 24 | **↓** | 0.112 | -0.047 | 0.271 | 75 | **↑** |
| El Salvador | -11.316 | 27 | **↓** | 0.387 | -0.772 | 1.559 | 52 | **↑** | -9.605 | 17 | **↓** | 0.152 | -0.238 | 0.543 | 63 | **↑** |
| Equatorial Guinea | -6.127 | 48 | **↓** | -0.339 | -0.420 | -0.258 | 44 | **↓** | 3.270 | 52 | **↑** | 0.203 | 0.142 | 0.265 | 57 | **↑** |
| Eritrea | 49.746 | 11 | **↑** | 1.769 | 1.523 | 2.016 | 7 | **↑** | 28.105 | 7 | **↑** | 1.016 | 0.801 | 1.232 | 5 | **↑** |
| Estonia | 3.105 | 92 | **↑** | -0.240 | -0.446 | -0.033 | 55 | **↓** | -2.219 | 51 | **↓** | 0.225 | 0.064 | 0.386 | 55 | **↑** |
| Ethiopia | 6.657 | 69 | **↑** | 0.004 | -0.434 | 0.443 | 86 | **↑** | -0.262 | 88 | **↓** | -0.096 | -0.310 | 0.118 | 38 | **↓** |
| Federated States of Micronesia | -11.580 | 24 | **↓** | -0.319 | -0.388 | -0.250 | 46 | **↓** | -0.094 | 92 | **↓** | -0.009 | -0.021 | 0.002 | 57 | **↓** |
| Fiji | -2.062 | 69 | **↓** | -0.033 | -0.049 | -0.016 | 98 | **↓** | -0.087 | 93 | **↓** | -0.002 | -0.015 | 0.011 | 62 | **↓** |
| Finland | 38.914 | 15 | **↑** | 0.453 | 0.214 | 0.692 | 47 | **↑** | 12.012 | 19 | **↑** | 0.288 | 0.123 | 0.453 | 51 | **↑** |
| France | 13.687 | 43 | **↑** | 0.486 | 0.342 | 0.631 | 46 | **↑** | 6.639 | 38 | **↑** | 0.339 | 0.241 | 0.438 | 47 | **↑** |
| Gabon | -12.508 | 20 | **↓** | -0.288 | -0.386 | -0.190 | 48 | **↓** | -9.614 | 16 | **↓** | -0.068 | -0.159 | 0.023 | 43 | **↓** |
| Georgia | 6.158 | 73 | **↑** | -0.007 | -0.134 | 0.119 | 104 | **↓** | -3.874 | 34 | **↓** | 0.092 | -0.005 | 0.188 | 88 | **↑** |
| Germany | 18.082 | 35 | **↑** | 0.533 | 0.330 | 0.736 | 42 | **↑** | 7.312 | 37 | **↑** | 0.406 | 0.261 | 0.551 | 40 | **↑** |
| Ghana | 48.406 | 12 | **↑** | 0.838 | 0.425 | 1.252 | 31 | **↑** | 24.858 | 11 | **↑** | 0.567 | 0.413 | 0.720 | 29 | **↑** |
| Greece | 3.751 | 88 | **↑** | 0.025 | -0.049 | 0.099 | 83 | **↑** | -1.974 | 59 | **↓** | -0.002 | -0.055 | 0.050 | 60 | **↓** |
| Greenland | -10.337 | 34 | **↓** | -0.506 | -0.667 | -0.345 | 33 | **↓** | 1.383 | 75 | **↑** | 0.066 | 0.057 | 0.075 | 98 | **↑** |
| Grenada | -3.024 | 62 | **↓** | -0.172 | -0.202 | -0.143 | 64 | **↓** | 3.383 | 51 | **↑** | 0.104 | 0.094 | 0.115 | 79 | **↑** |
| Guam | -5.035 | 51 | **↓** | -0.018 | -0.082 | 0.045 | 103 | **↓** | -0.294 | 86 | **↓** | -0.021 | -0.036 | -0.005 | 53 | **↓** |
| Guatemala | 7.818 | 62 | **↑** | 1.020 | 0.678 | 1.363 | 17 | **↑** | 13.156 | 17 | **↑** | 0.937 | 0.723 | 1.151 | 7 | **↑** |
| Guinea | -24.752 | 10 | **↓** | -0.661 | -0.854 | -0.468 | 27 | **↓** | -13.232 | 8 | **↓** | -0.287 | -0.440 | -0.134 | 25 | **↓** |
| Guinea-Bissau | 40.254 | 14 | **↑** | 0.896 | 0.614 | 1.180 | 28 | **↑** | 32.168 | 3 | **↑** | 0.036 | 0.650 | 1.002 | 116 | **↑** |
| Guyana | -3.131 | 61 | **↓** | -0.814 | -1.160 | -0.467 | 21 | **↓** | 3.519 | 49 | **↑** | -0.292 | -0.517 | -0.067 | 23 | **↓** |
| Haiti | -21.998 | 11 | **↓** | -0.408 | -0.653 | -0.164 | 39 | **↓** | -1.240 | 67 | **↓** | 0.265 | 0.100 | 0.430 | 54 | **↑** |
| Honduras | -2.078 | 68 | **↓** | 0.095 | -1.056 | 1.260 | 74 | **↑** | 2.626 | 61 | **↑** | 0.344 | -0.307 | 1.000 | 46 | **↑** |
| Hungary | 12.968 | 45 | **↑** | 0.171 | -0.033 | 0.374 | 64 | **↑** | -7.904 | 23 | **↓** | -0.085 | -0.231 | 0.060 | 39 | **↓** |
| Iceland | 0.366 | 115 | **↑** | -0.105 | -0.170 | -0.039 | 79 | **↓** | -0.699 | 77 | **↓** | 0.047 | -0.008 | 0.102 | 112 | **↑** |
| India | 27.219 | 21 | **↑** | 0.791 | 0.447 | 1.136 | 35 | **↑** | 12.752 | 18 | **↑** | 0.347 | 0.162 | 0.534 | 45 | **↑** |
| Indonesia | 56.689 | 8 | **↑** | 1.673 | 0.981 | 2.370 | 9 | **↑** | 30.680 | 6 | **↑** | 0.845 | 0.397 | 1.296 | 13 | **↑** |
| Iran | 6.075 | 74 | **↑** | -0.235 | -0.601 | 0.134 | 57 | **↓** | -0.664 | 78 | **↓** | -0.130 | -0.269 | 0.009 | 34 | **↓** |
| Iraq | 4.980 | 82 | **↑** | 0.102 | 0.023 | 0.182 | 73 | **↑** | -0.411 | 84 | **↓** | 0.047 | 0.007 | 0.087 | 111 | **↑** |
| Ireland | 3.796 | 87 | **↑** | -0.003 | -0.081 | 0.076 | 107 | **↓** | -2.435 | 48 | **↓** | 0.027 | -0.035 | 0.090 | 119 | **↑** |
| Israel | -1.812 | 72 | **↓** | -0.158 | -0.267 | -0.049 | 70 | **↓** | 0.601 | 81 | **↑** | 0.053 | 0.017 | 0.089 | 108 | **↑** |
| Italy | 17.912 | 36 | **↑** | 0.288 | -0.225 | 0.804 | 56 | **↑** | 10.140 | 24 | **↑** | 0.398 | 0.100 | 0.696 | 41 | **↑** |
| Jamaica | -1.750 | 73 | **↓** | -0.127 | -0.154 | -0.101 | 74 | **↓** | 2.602 | 63 | **↑** | 0.064 | 0.049 | 0.078 | 103 | **↑** |
| Japan | 9.365 | 58 | **↑** | 0.002 | -0.216 | 0.220 | 87 | **↑** | -3.121 | 37 | **↓** | 0.095 | -0.041 | 0.231 | 86 | **↑** |
| Jordan | 58.164 | 7 | **↑** | 1.018 | 0.568 | 1.469 | 18 | **↑** | 6.553 | 40 | **↑** | 0.152 | -0.133 | 0.438 | 62 | **↑** |
| Kazakhstan | 12.795 | 47 | **↑** | 0.937 | 0.620 | 1.255 | 23 | **↑** | -2.033 | 55 | **↓** | 0.548 | 0.305 | 0.792 | 30 | **↑** |
| Kenya | 80.848 | 3 | **↑** | 1.832 | 0.870 | 2.804 | 6 | **↑** | 25.591 | 9 | **↑** | 0.614 | 0.210 | 1.019 | 24 | **↑** |
| Kiribati | -7.344 | 39 | **↓** | -0.225 | -0.258 | -0.192 | 58 | **↓** | -0.100 | 91 | **↓** | 0.002 | -0.013 | 0.016 | 130 | **↑** |
| Kuwait | 7.026 | 64 | **↑** | 0.158 | 0.091 | 0.225 | 67 | **↑** | -2.090 | 54 | **↓** | -0.036 | -0.063 | -0.010 | 51 | **↓** |
| Kyrgyzstan | -1.356 | 74 | **↓** | 0.161 | -0.074 | 0.396 | 65 | **↑** | -8.831 | 21 | **↓** | 0.106 | -0.071 | 0.284 | 78 | **↑** |
| Laos | -6.973 | 42 | **↓** | -0.151 | -0.266 | -0.036 | 73 | **↓** | 0.690 | 79 | **↑** | 0.023 | 0.016 | 0.029 | 122 | **↑** |
| Latvia | 22.135 | 28 | **↑** | 0.640 | 0.428 | 0.852 | 37 | **↑** | 9.629 | 27 | **↑** | 0.892 | 0.663 | 1.122 | 9 | **↑** |
| Lebanon | 6.795 | 67 | **↑** | 1.114 | 0.789 | 1.440 | 13 | **↑** | -2.496 | 44 | **↓** | 0.641 | 0.390 | 0.892 | 20 | **↑** |
| Lesotho | -3.567 | 57 | **↓** | -1.534 | -2.638 | -0.418 | 12 | **↓** | -0.619 | 79 | **↓** | -0.765 | -1.451 | -0.075 | 11 | **↓** |
| Liberia | -27.251 | 9 | **↓** | -1.718 | -2.031 | -1.405 | 10 | **↓** | -15.024 | 7 | **↓** | -0.989 | -1.252 | -0.725 | 8 | **↓** |
| Libya | 27.223 | 20 | **↑** | 0.957 | 0.818 | 1.095 | 22 | **↑** | 2.812 | 60 | **↑** | 0.328 | 0.184 | 0.472 | 49 | **↑** |
| Lithuania | 26.191 | 22 | **↑** | 0.803 | 0.597 | 1.009 | 33 | **↑** | 3.837 | 46 | **↑** | 0.605 | 0.396 | 0.815 | 25 | **↑** |
| Luxembourg | -0.793 | 79 | **↓** | -0.089 | -0.175 | -0.003 | 82 | **↓** | 0.591 | 82 | **↑** | 0.067 | 0.032 | 0.103 | 95 | **↑** |
| Macedonia | 13.499 | 44 | **↑** | 0.106 | -0.128 | 0.341 | 71 | **↑** | -10.586 | 13 | **↓** | -0.061 | -0.239 | 0.118 | 47 | **↓** |
| Madagascar | 11.581 | 51 | **↑** | -0.607 | -1.399 | 0.192 | 32 | **↓** | 10.180 | 23 | **↑** | -0.120 | -0.524 | 0.286 | 36 | **↓** |
| Malawi | -39.402 | 5 | **↓** | -2.933 | -4.463 | -1.378 | 4 | **↓** | -29.058 | 4 | **↓** | -1.684 | -2.521 | -0.840 | 4 | **↓** |
| Malaysia | 1.232 | 108 | **↑** | -0.051 | -0.101 | -0.001 | 93 | **↓** | -1.188 | 68 | **↓** | 0.087 | 0.015 | 0.160 | 91 | **↑** |
| Maldives | -1.916 | 71 | **↓** | -0.417 | -0.727 | -0.107 | 37 | **↓** | 0.112 | 98 | **↑** | -0.057 | -0.123 | 0.009 | 48 | **↓** |
| Mali | -2.776 | 64 | **↓** | 0.233 | -0.070 | 0.537 | 60 | **↑** | 8.970 | 31 | **↑** | 0.605 | 0.384 | 0.826 | 26 | **↑** |
| Malta | 2.348 | 97 | **↑** | -0.066 | -0.145 | 0.014 | 87 | **↓** | -1.362 | 64 | **↓** | 0.029 | -0.024 | 0.082 | 117 | **↑** |
| Marshall Islands | -10.641 | 30 | **↓** | -0.246 | -0.330 | -0.162 | 52 | **↓** | -0.293 | 87 | **↓** | -0.014 | -0.026 | -0.001 | 56 | **↓** |
| Mauritania | -14.055 | 17 | **↓** | -0.406 | -0.537 | -0.274 | 40 | **↓** | 2.094 | 69 | **↑** | 0.826 | 0.001 | 0.072 | 14 | **↑** |
| Mauritius | 0.320 | 116 | **↑** | -0.062 | -0.118 | -0.006 | 88 | **↓** | -0.333 | 85 | **↓** | 0.097 | 0.038 | 0.157 | 85 | **↑** |
| Mexico | 67.825 | 5 | **↑** | 0.493 | 0.128 | 0.859 | 45 | **↑** | 37.949 | 1 | **↑** | 0.492 | 0.192 | 0.792 | 32 | **↑** |
| Moldova | 4.154 | 85 | **↑** | -0.449 | -0.976 | 0.081 | 35 | **↓** | -2.380 | 49 | **↓** | -0.144 | -0.332 | 0.043 | 33 | **↓** |
| Mongolia | 1.914 | 103 | **↑** | -0.109 | -0.206 | -0.012 | 78 | **↓** | 0.346 | 89 | **↑** | 0.102 | 0.062 | 0.141 | 80 | **↑** |
| Montenegro | 12.834 | 46 | **↑** | 0.145 | -0.025 | 0.315 | 68 | **↑** | -8.966 | 20 | **↓** | -0.052 | -0.184 | 0.081 | 49 | **↓** |
| Morocco | 43.198 | 13 | **↑** | 2.772 | 1.509 | 4.051 | 3 | **↑** | 25.306 | 10 | **↑** | 1.671 | 0.987 | 2.360 | 2 | **↑** |
| Mozambique | -6.146 | 47 | **↓** | -1.752 | -2.408 | -1.092 | 9 | **↓** | 3.019 | 55 | **↑** | -0.879 | -1.319 | -0.438 | 9 | **↓** |
| Myanmar | -7.319 | 40 | **↓** | -0.215 | -0.294 | -0.136 | 59 | **↓** | 1.104 | 76 | **↑** | 0.023 | 0.013 | 0.034 | 121 | **↑** |
| Namibia | -68.973 | 2 | **↓** | -5.842 | -6.623 | -5.054 | 1 | **↓** | -38.571 | 3 | **↓** | -2.216 | -2.532 | -1.898 | 2 | **↓** |
| Nepal | -8.960 | 35 | **↓** | -0.002 | -1.110 | 1.118 | 108 | **↓** | 0.851 | 77 | **↑** | -0.291 | -0.885 | 0.306 | 24 | **↓** |
| Netherlands | 1.966 | 102 | **↑** | -0.091 | -0.165 | -0.017 | 81 | **↓** | -0.765 | 75 | **↓** | 0.077 | 0.026 | 0.127 | 93 | **↑** |
| New Zealand | 18.837 | 31 | **↑** | 1.006 | 0.795 | 1.218 | 19 | **↑** | 3.540 | 48 | **↑** | 0.304 | 0.212 | 0.395 | 50 | **↑** |
| Nicaragua | -2.406 | 66 | **↓** | 0.980 | 0.345 | 1.619 | 20 | **↑** | 1.794 | 71 | **↑** | 0.640 | 0.372 | 0.908 | 21 | **↑** |
| Niger | -49.673 | 3 | **↓** | -2.747 | -3.037 | -2.457 | 5 | **↓** | -38.985 | 2 | **↓** | -1.843 | -1.988 | -1.697 | 3 | **↓** |
| Nigeria | 5.458 | 78 | **↑** | -0.978 | -1.760 | -0.189 | 17 | **↓** | 2.544 | 64 | **↑** | -0.849 | -1.412 | -0.284 | 10 | **↓** |
| North Korea | 0.859 | 110 | **↑** | -0.023 | -0.060 | 0.014 | 100 | **↓** | -2.159 | 53 | **↓** | -0.042 | -0.061 | -0.023 | 50 | **↓** |
| Northern Mariana Islands | -6.842 | 43 | **↓** | -0.236 | -0.260 | -0.211 | 56 | **↓** | -0.072 | 94 | **↓** | -0.016 | -0.033 | 0.001 | 54 | **↓** |
| Norway | 9.155 | 59 | **↑** | 0.376 | 0.314 | 0.438 | 53 | **↑** | 0.147 | 97 | **↑** | 0.020 | 0.006 | 0.034 | 124 | **↑** |
| Oman | 12.256 | 48 | **↑** | 0.335 | 0.182 | 0.489 | 55 | **↑** | -6.686 | 27 | **↓** | -0.198 | -0.306 | -0.089 | 29 | **↓** |
| Pakistan | -30.747 | 8 | **↓** | -1.575 | -1.926 | -1.223 | 11 | **↓** | -10.826 | 12 | **↓** | -0.653 | -0.905 | -0.401 | 13 | **↓** |
| Palestine | 6.887 | 66 | **↑** | 0.133 | 0.015 | 0.252 | 70 | **↑** | -2.277 | 50 | **↓** | 0.009 | -0.066 | 0.083 | 128 | **↑** |
| Panama | -3.229 | 60 | **↓** | -0.126 | -0.160 | -0.093 | 75 | **↓** | 2.900 | 57 | **↑** | 0.142 | 0.117 | 0.167 | 66 | **↑** |
| Papua New Guinea | -12.436 | 21 | **↓** | -0.278 | -0.424 | -0.132 | 49 | **↓** | 0.033 | 101 | **↑** | -0.007 | -0.018 | 0.005 | 58 | **↓** |
| Paraguay | 22.598 | 26 | **↑** | 0.914 | 0.380 | 1.451 | 26 | **↑** | 27.710 | 8 | **↑** | 0.872 | 0.632 | 1.112 | 10 | **↑** |
| Peru | 30.190 | 19 | **↑** | 3.659 | 2.270 | 5.066 | 2 | **↑** | 31.553 | 5 | **↑** | 2.091 | 1.609 | 2.576 | 1 | **↑** |
| Philippines | 0.390 | 114 | **↑** | -0.152 | -0.454 | 0.152 | 72 | **↓** | 10.986 | 21 | **↑** | 0.411 | 0.156 | 0.667 | 39 | **↑** |
| Poland | 34.028 | 17 | **↑** | 0.908 | 0.716 | 1.100 | 27 | **↑** | 0.174 | 94 | **↑** | 0.362 | 0.104 | 0.620 | 43 | **↑** |
| Portugal | 2.079 | 101 | **↑** | -0.060 | -0.132 | 0.012 | 90 | **↓** | -0.614 | 80 | **↓** | 0.051 | -0.002 | 0.104 | 109 | **↑** |
| Puerto Rico | 3.369 | 89 | **↑** | 0.026 | -0.012 | 0.064 | 82 | **↑** | -0.138 | 90 | **↓** | 0.020 | -0.018 | 0.059 | 123 | **↑** |
| Qatar | 9.679 | 57 | **↑** | 0.243 | 0.057 | 0.428 | 59 | **↑** | -4.771 | 31 | **↓** | -0.105 | -0.264 | 0.054 | 37 | **↓** |
| Romania | 7.360 | 63 | **↑** | -0.080 | -0.241 | 0.080 | 84 | **↓** | -3.817 | 35 | **↓** | 0.160 | 0.033 | 0.286 | 61 | **↑** |
| Russian Federation | 6.252 | 72 | **↑** | -0.034 | -0.154 | 0.087 | 97 | **↓** | -3.011 | 38 | **↓** | 0.112 | 0.016 | 0.209 | 76 | **↑** |
| Rwanda | 31.454 | 18 | **↑** | -0.632 | -2.091 | 0.849 | 31 | **↓** | 3.150 | 53 | **↑** | -0.465 | -0.849 | -0.080 | 16 | **↓** |
| Saint Lucia | -3.344 | 59 | **↓** | -0.202 | -0.237 | -0.166 | 63 | **↓** | 3.544 | 47 | **↑** | 0.117 | 0.106 | 0.127 | 73 | **↑** |
| Saint Vincent and the Grenadines | -1.205 | 76 | **↓** | -0.166 | -0.206 | -0.126 | 66 | **↓** | 2.816 | 59 | **↑** | 0.087 | 0.079 | 0.096 | 90 | **↑** |
| Samoa | -5.269 | 50 | **↓** | -0.166 | -0.183 | -0.148 | 67 | **↓** | 0.171 | 95 | **↑** | 0.008 | 0.000 | 0.017 | 129 | **↑** |
| Sao Tome and Principe | 5.452 | 79 | **↑** | -0.808 | -1.529 | -0.082 | 23 | **↓** | -3.890 | 33 | **↓** | -0.292 | -0.412 | -0.172 | 22 | **↓** |
| Saudi Arabia | 10.280 | 54 | **↑** | 0.222 | 0.066 | 0.377 | 62 | **↑** | -5.251 | 30 | **↓** | -0.065 | -0.186 | 0.057 | 45 | **↓** |
| Senegal | -10.477 | 31 | **↓** | -0.168 | -0.438 | 0.104 | 65 | **↓** | -1.411 | 63 | **↓** | 0.333 | -0.021 | 0.688 | 48 | **↑** |
| Serbia | 18.425 | 33 | **↑** | 0.278 | 0.073 | 0.483 | 57 | **↑** | -11.455 | 10 | **↓** | -0.156 | -0.302 | -0.010 | 32 | **↓** |
| Seychelles | 3.096 | 93 | **↑** | 0.086 | 0.061 | 0.110 | 76 | **↑** | -2.503 | 43 | **↓** | -0.074 | -0.104 | -0.044 | 41 | **↓** |
| Sierra Leone | -35.378 | 7 | **↓** | -1.923 | -2.273 | -1.572 | 8 | **↓** | -26.152 | 5 | **↓** | -1.134 | -1.453 | -0.814 | 5 | **↓** |
| Singapore | -10.437 | 32 | **↓** | -0.459 | -0.723 | -0.194 | 34 | **↓** | -1.273 | 65 | **↓** | 0.147 | -0.013 | 0.308 | 64 | **↑** |
| Slovakia | 17.723 | 37 | **↑** | 0.362 | 0.186 | 0.539 | 54 | **↑** | -11.447 | 11 | **↓** | -0.198 | -0.335 | -0.060 | 28 | **↓** |
| Slovenia | 92.445 | 2 | **↑** | 2.603 | 2.017 | 3.193 | 5 | **↑** | -5.644 | 29 | **↓** | 0.418 | 0.117 | 0.719 | 37 | **↑** |
| Solomon Islands | -13.399 | 19 | **↓** | -0.344 | -0.477 | -0.211 | 43 | **↓** | 0.099 | 100 | **↑** | 0.000 | -0.012 | 0.012 | 132 | **↑** |
| Somalia | -3.726 | 56 | **↓** | -0.093 | -0.127 | -0.060 | 80 | **↓** | 1.998 | 70 | **↑** | 0.063 | 0.050 | 0.076 | 104 | **↑** |
| South Africa | -1.201 | 77 | **↓** | 0.507 | 0.257 | 0.756 | 43 | **↑** | -1.070 | 70 | **↓** | 0.445 | 0.252 | 0.640 | 35 | **↑** |
| South Korea | -13.980 | 18 | **↓** | -0.436 | -0.662 | -0.211 | 36 | **↓** | -0.457 | 83 | **↓** | 0.089 | 0.025 | 0.154 | 89 | **↑** |
| South Sudan | -12.157 | 22 | **↓** | -0.377 | -0.482 | -0.271 | 41 | **↓** | 2.609 | 62 | **↑** | 0.078 | 0.056 | 0.100 | 92 | **↑** |
| Spain | 25.155 | 23 | **↑** | 0.885 | 0.729 | 1.041 | 29 | **↑** | 8.540 | 32 | **↑** | 0.449 | 0.329 | 0.570 | 34 | **↑** |
| Sri Lanka | 37.614 | 16 | **↑** | 0.452 | 0.222 | 0.683 | 48 | **↑** | 7.765 | 36 | **↑** | 0.028 | -0.125 | 0.181 | 118 | **↑** |
| Sudan | 22.682 | 25 | **↑** | 0.880 | 0.558 | 1.203 | 30 | **↑** | 17.777 | 13 | **↑** | 0.685 | 0.467 | 0.904 | 17 | **↑** |
| Suriname | 0.925 | 109 | **↑** | -0.057 | -0.091 | -0.024 | 91 | **↓** | 1.434 | 74 | **↑** | 0.065 | 0.057 | 0.074 | 101 | **↑** |
| Swaziland | -3.752 | 55 | **↓** | -0.987 | -1.523 | -0.449 | 16 | **↓** | -0.882 | 74 | **↓** | -0.361 | -0.582 | -0.139 | 20 | **↓** |
| Sweden | 2.975 | 95 | **↑** | -0.040 | -0.143 | 0.063 | 95 | **↓** | -2.998 | 39 | **↓** | 0.013 | -0.086 | 0.112 | 126 | **↑** |
| Switzerland | -3.972 | 54 | **↓** | -0.214 | -0.365 | -0.063 | 60 | **↓** | 0.266 | 91 | **↑** | 0.098 | 0.030 | 0.166 | 83 | **↑** |
| Syria | 22.299 | 27 | **↑** | 1.071 | 0.561 | 1.584 | 15 | **↑** | 5.329 | 43 | **↑** | 0.503 | 0.276 | 0.732 | 31 | **↑** |
| Taiwan | -2.910 | 63 | **↓** | -0.247 | -0.326 | -0.168 | 51 | **↓** | -2.441 | 46 | **↓** | 0.115 | 0.021 | 0.210 | 74 | **↑** |
| Tajikistan | -11.369 | 26 | **↓** | -0.822 | -1.033 | -0.611 | 20 | **↓** | -7.147 | 26 | **↓** | -0.312 | -0.374 | -0.250 | 21 | **↓** |
| Tanzania | 6.592 | 71 | **↑** | 0.779 | 0.263 | 1.297 | 36 | **↑** | 8.256 | 33 | **↑** | 0.691 | 0.313 | 1.071 | 16 | **↑** |
| Thailand | 23.734 | 24 | **↑** | 0.144 | -0.084 | 0.372 | 69 | **↑** | 11.948 | 20 | **↑** | 0.269 | 0.159 | 0.380 | 53 | **↑** |
| The Bahamas | -5.426 | 49 | **↓** | -0.243 | -0.279 | -0.208 | 53 | **↓** | 3.413 | 50 | **↑** | 0.100 | 0.087 | 0.114 | 81 | **↑** |
| The Gambia | -16.909 | 14 | **↓** | -1.007 | -1.228 | -0.786 | 15 | **↓** | -10.467 | 14 | **↓** | -0.554 | -0.689 | -0.420 | 15 | **↓** |
| Timor-Leste | -8.530 | 37 | **↓** | -0.838 | -1.097 | -0.579 | 19 | **↓** | 1.510 | 73 | **↑** | -0.361 | -0.617 | -0.104 | 19 | **↓** |
| Togo | 19.790 | 30 | **↑** | -0.210 | -0.720 | 0.302 | 62 | **↓** | 9.736 | 26 | **↑** | 0.144 | -0.112 | 0.401 | 65 | **↑** |
| Tonga | 1.255 | 107 | **↑** | 0.024 | 0.000 | 0.049 | 84 | **↑** | -0.993 | 71 | **↓** | -0.035 | -0.045 | -0.025 | 52 | **↓** |
| Trinidad and Tobago | 18.341 | 34 | **↑** | 0.178 | 0.033 | 0.323 | 63 | **↑** | 4.027 | 44 | **↑** | 0.124 | 0.028 | 0.220 | 69 | **↑** |
| Tunisia | 62.249 | 6 | **↑** | 1.185 | 0.734 | 1.639 | 12 | **↑** | 17.553 | 14 | **↑** | 0.683 | 0.463 | 0.904 | 18 | **↑** |
| Turkey | 104.936 | 1 | **↑** | 3.667 | 3.108 | 4.228 | 1 | **↑** | 23.512 | 12 | **↑** | 1.326 | 1.041 | 1.611 | 3 | **↑** |
| Turkmenistan | 6.024 | 75 | **↑** | -0.037 | -0.168 | 0.095 | 96 | **↓** | -2.802 | 40 | **↓** | 0.072 | -0.030 | 0.174 | 94 | **↑** |
| Uganda | -15.820 | 16 | **↓** | -1.965 | -3.406 | -0.502 | 7 | **↓** | -9.213 | 18 | **↓** | -1.065 | -1.755 | -0.370 | 7 | **↓** |
| Ukraine | 4.568 | 84 | **↑** | 0.811 | 0.361 | 1.263 | 32 | **↑** | -2.734 | 41 | **↓** | 0.491 | 0.257 | 0.726 | 33 | **↑** |
| United Arab Emirates | 14.086 | 42 | **↑** | 0.413 | 0.210 | 0.617 | 50 | **↑** | -8.040 | 22 | **↓** | -0.220 | -0.375 | -0.065 | 27 | **↓** |
| United Kingdom | -2.467 | 65 | **↓** | 0.070 | -0.139 | 0.279 | 80 | **↑** | -1.820 | 61 | **↓** | 0.067 | -0.014 | 0.148 | 97 | **↑** |
| United States | 5.745 | 77 | **↑** | -0.812 | -1.972 | 0.361 | 22 | **↓** | 0.715 | 78 | **↑** | -0.414 | -0.683 | -0.144 | 18 | **↓** |
| Uruguay | -19.637 | 13 | **↓** | -0.757 | -0.845 | -0.668 | 26 | **↓** | 0.357 | 88 | **↑** | 0.040 | 0.031 | 0.049 | 113 | **↑** |
| Uzbekistan | 8.261 | 61 | **↑** | 0.799 | 0.499 | 1.100 | 34 | **↑** | -1.967 | 60 | **↓** | 0.376 | 0.199 | 0.553 | 42 | **↑** |
| Vanuatu | -8.797 | 36 | **↓** | -0.243 | -0.356 | -0.130 | 54 | **↓** | 0.163 | 96 | **↑** | -0.001 | -0.014 | 0.012 | 63 | **↓** |
| Venezuela | -7.204 | 41 | **↓** | -0.310 | -0.358 | -0.262 | 47 | **↓** | 2.821 | 58 | **↑** | 0.187 | 0.154 | 0.220 | 60 | **↑** |
| Vietnam | -7.853 | 38 | **↓** | 1.048 | 0.474 | 1.624 | 16 | **↑** | 0.428 | 86 | **↑** | 0.631 | 0.418 | 0.845 | 22 | **↑** |
| Virgin Islands, U.S. | -6.219 | 46 | **↓** | -0.355 | -0.415 | -0.294 | 42 | **↓** | 3.086 | 54 | **↑** | 0.120 | 0.109 | 0.132 | 71 | **↑** |
| Yemen | -6.386 | 45 | **↓** | 0.229 | -0.306 | 0.767 | 61 | **↑** | -1.261 | 66 | **↓** | 0.136 | -0.152 | 0.425 | 68 | **↑** |
| Zambia | -71.816 | 1 | **↓** | -5.783 | -6.415 | -5.147 | 2 | **↓** | -47.723 | 1 | **↓** | -2.863 | -3.171 | -2.553 | 1 | **↓** |
| Zimbabwe | 11.419 | 52 | **↑** | -0.060 | -1.105 | 0.996 | 89 | **↓** | 9.031 | 30 | **↑** | -0.177 | -0.706 | 0.355 | 30 | **↓** |

a: percent change.

b: annual percent change

c: confidence interval
